# The Rise of Heatstroke as a Method of Depopulating Pigs and Poultry: Implications for the US Veterinary Profession

**DOI:** 10.3390/ani13010140

**Published:** 2022-12-29

**Authors:** Gwendolen Reyes-Illg, Jessica E. Martin, Indu Mani, James Reynolds, Barry Kipperman

**Affiliations:** 1 Farm Animal Program, Animal Welfare Institute, Washington, DC 20003, USA; 2 School of Natural and Environmental Sciences, Newcastle University, Newcastle upon Tyne NE1 7RU, UK; 3 Brief Media, Tulsa, OK 74104, USA; 4 College of Veterinary Medicine, Western University of Health Sciences, Pomona, CA 91766, USA; 5 School of Veterinary Medicine, University of California, Davis, CA 95616, USA

**Keywords:** on-farm emergency killing, livestock, farm animal, veterinary ethics, ventilation shutdown (VSD), swine, animal welfare, animal-derived foods (ADF), euthanasia, highly pathogenic avian influenza (HPAI)

## Abstract

**Simple Summary:**

In response to disease outbreaks or other urgent circumstances, an increasing number of farm animals in the United States (US) are being killed en masse by depopulation. In the past few years, depopulation methods that rely on heatstroke as the mechanism of killing have been used with increasing frequency to kill birds and pigs raised for food production. While they are defended as expedient and faster to implement, heatstroke-based methods severely compromise animal welfare and there is a prolonged period prior to the animals losing consciousness. The US veterinary profession is entrusted with an ethical responsibility to protect and advance animal welfare, yet its classification of the heatstroke-based depopulation method Ventilation Shutdown Plus is used to justify this method’s widespread use. Numerous strategies are suggested for how the US veterinary profession, including the American Veterinary Medical Association, can encourage the use of more humane methods when depopulations are performed.

**Abstract:**

Depopulation of food-producing animals is becoming increasingly common in response to both disease outbreaks and supply chain disruptions. In 2019, the American Veterinary Medical Association released depopulation guidelines classifying certain heatstroke-based killing methods as “permitted in constrained circumstances”, when circumstances of the emergency constrain reasonable implementation of “preferred” methods. Since then, tens of millions of birds and pigs have been killed by such methods, termed ventilation shutdown (VSD) Plus Heat and VSD Plus High Temperature and Humidity. While no research using validated measures of animal welfare assessment has been performed on these methods, their pathophysiology suggests that animals are likely to experience pain, anxiety, nausea, and heat distress prior to loss of consciousness. Heatstroke-based methods may result in prolonged suffering and often do not achieve 100% mortality. Potential and available alternative depopulation methods are briefly reviewed. The veterinary profession’s ethical obligation to protect animal welfare in the context of depopulations is discussed.

## 1. Introduction

The intentional mass killing (i.e., depopulation) of farm animals has long been employed for disease control, however the methods utilized have been neglected in terms of scientific validation, especially in relation to optimizing animal welfare. This is exacerbated by developments in the field often being a “reactive” response to an on-going crisis. For example, in 2020, the coronavirus (COVID-19) pandemic led to unprecedented supply chain disruption in the United States (US) [1,2], resulting in hundreds of thousands of pigs being depopulated by a newly developed method, “ventilation shutdown plus” (VSD+), which causes death by heatstroke. Similarly, many millions of chickens, turkeys, and ducks infected with or at risk of exposure to Highly Pathogenic Avian Influenza (HPAI) have been depopulated with VSD+ in 2022 [3,4]. Prior to this, “ventilation shutdown” (VSD) was used four times during the HPAI outbreak that began in December 2014 and resulted in the deaths of 43 million chickens and 7.4 million turkeys over the following 14 months [5,6].

Heatstroke is characterized by nonpyrogenic hyperthermia accompanied by a systemic inflammatory response that often causes organ failure and death [7]. In veterinary medicine, its impact on animal health and welfare is well recognized, such that the veterinary community works to protect livestock from heat stress [8,9,10] and decrease the incidence of heatstroke in canine and feline patient populations [11,12]. Because death by heatstroke is likely to involve prolonged suffering, its use as a method of killing farm animals en masse in response to disease or other urgent circumstances has generated significant controversy within the global veterinary profession [13,14,15,16,17]. The recent sharp rise in use of heatstroke-based methods was preceded by the official classification by the American Veterinary Medical Association (AVMA) in 2019 of some of these methods as “permitted in constrained circumstances”, i.e., circumstances that constrain the ability to use methods classified as “preferred” [18]. Perceived “AVMA approval” of these methods, coupled with the scale of HPAI- and COVID-19-related depopulations, has contributed to the rise in their use.

The number of birds depopulated in the US in 2022 as of November (52.5 million) is equivalent to well over half of the country’s pet dog population [4,19]. The selection and development of depopulation methods is complex and not always amenable to clear and simple strategies, with conflicting priorities for animal welfare, human safety, biosecurity, cost, equipment/resource availability, and operational factors. In addition, the emotional impact of depopulating animals on veterinarians and other depopulation workers has been studied as a major human well-being concern since 2001 [20,21,22,23,24,25,26,27].

All these factors highlight the need for the veterinary profession to examine its ethical responsibilities regarding animal depopulations, including its role in assessing depopulation methods and promoting the use of methods consistent with the profession’s accepted ethical principles. Understanding the context in which depopulations occur and the factors that affect which depopulation methods are used is essential for this endeavor.

### 1.1. Depopulation Defined

Depopulation is defined as the mass killing of large numbers of animals, typically occurring in response to urgent or emergency situations, including infectious disease outbreaks and both natural and manmade disasters [18]. A depopulation typically refers to the killing of all animals residing at a single location, such as a farm.

In 2019, the AVMA published its first edition of the *Guidelines for the Depopulation of Animals* (hereafter referred to as the *Guidelines*) [18]. The *Guidelines* include general guidance on depopulation along with sections stratified by species or animal group. Each section is written by a specific working group comprised of veterinarians and associated professionals. The *Guidelines* describe common reasons for depopulation, methods, and special considerations for each species. They allow for the destruction of both healthy and sick animals, when required. The *Guidelines* state that the AVMA’s guidance documents on euthanasia or slaughter, rather than depopulation, should be used for “precautionary killing” or “prophylactic culling”. These terms typically refer to killing of healthy animals to prevent spread of disease [28], but are left undefined in the *Guidelines*.

Although animal welfare, psychological impact on workers implementing depopulation, and public trust are all posited to be important concerns, rapid response to the emergency is often the overarching consideration. Human health considerations may be paramount, especially in the event of highly zoonotic disease. Specifically, the *Guidelines* state that during depopulations, animal welfare should be afforded as much consideration as is “practicable”, but “rapid destruction … in response to urgent circumstances” is a primary consideration; death may not be “painless and distress free.”

Depopulation methods are classified as *preferred*, *permitted in constrained circumstances*, and *not recommended*. Per the *Guidelines*, *preferred* methods should be used in creating emergency response plans and “when circumstances allow reasonable implementation.” Methods classified as *permitted in constrained circumstances* are allowed when constraints, such as “zoonotic disease response time, depopulation efficiency, deployable resources, equipment, animal access, disruption of infrastructure, and disease transmission risk” constrain the ability to use *preferred* methods. A method classified as *not recommended* should only be used when methods in the other two categories cannot be “reasonably implemented” and failing to intervene is likely to cause more animal suffering than using the method. Of note, AVMA representatives have stated that the *not recommended* classification does not mean “unacceptable” [29]. The *Guidelines* state that “the use of less preferred methods should not become synonymous with standard practice”.

### 1.2. Historical Factors Leading to the Development of Depopulation Methods

For centuries, methods to control or eradicate farm animal diseases of zoonotic and economic importance have included killing affected animals to limit pathogen spread [30]. Prior to the major intensification of animal production, farms traditionally housed small numbers of animals in extensive housing systems. When diseases required on-farm killing, this was done by killing individual animals, mainly by gunshot (livestock) and decapitation or cervical dislocation (poultry) [31,32].

Today, the number of pig and poultry farms in the US is only between two and six percent of the number that existed in 1950 [33,34], even as the growth of the US and global populations has contributed to an increase in demand for animal-derived foods (ADF), and thus the number of animals used in food production. Over 127 million pigs and 9 billion chickens are now slaughtered each year in the US and nearly 400 million hens are used for egg production [35,36]. Many hog and poultry farms house thousands to hundreds of thousands of animals in each barn/shed [18,37,38]. Slaughter facilities consolidated simultaneously with livestock farms. In 1968, there were over 9000 livestock slaughter plants in the US, but by 2021, there were fewer than 2800. [36,39]. Fewer slaughter plants processing more animals per plant means animals are transported longer distances from farms and scheduling for slaughter loads is carefully managed to ensure continuous operation [40,41].

Any disruption in the supply chain that leads to pigs or poultry not being slaughtered at the designated time leads to an immediate bottleneck [40]. This can lead to pressure to depopulate rapidly, since the “slaughter-weight” animals continue to grow despite lack of pen or barn space, and the next batch of animals is already in the production pipeline. However, the methods historically used for disease control-associated on-farm killing are often unfeasible given the size of modern facilities and the number of animals involved. This has led to development of methods of “mass killing”, or depopulation, that typically kill animals as a large group.

### 1.3. Recent Depopulations

In 2020, COVID-19 disrupted US supply chains and major outbreaks among workers temporarily closed slaughterhouses [40,41]. This led to over-crowding on farms and strained supply and demand linkages, ultimately resulting in mass killing of large numbers of pigs and chickens, usually on-farm [1,42]. Animal welfare concerns secondary to overcrowding and feed shortages were cited as key justification for these depopulations, however, economic factors also played a role, as feed requirements increased and animals grew too large to be slaughtered at the processing plants typically used [1,40,42,43,44,45].

In February 2022, HPAI was identified in the US, where a “stamping out” policy dictates that all birds on any premise in which HPAI is detected must be killed, ideally within 48 h of a presumptive diagnosis [46].

## 2. Description of Heatstroke-Based Depopulation Methods

While depopulation methods relying on heatstroke are not recognized by the World Organization for Animal Health (WOAH, formerly known by its French acronym OIE) Terrestrial Code [47], they are discussed in the AVMA *Guidelines* in the sections on Swine and Poultry [18]. The *Guidelines* describe ventilation shutdown as “closing up the house, shutting inlets, and turning off the fans”, and allowing body heat from the animals to raise the temperature in the house until the animals die from hyperthermia. The *Guidelines* also discuss “Ventilation Shutdown Plus” (VSD+), where the “plus” refers to the addition of heat or carbon dioxide (CO_2_), to hasten the killing process. The *Guidelines* categorize VSD+ as “permitted in constrained circumstances” for poultry confined in buildings (either floor-reared or in cages), ratites, and pigs, while VSD alone is categorized as “not recommended” for all poultry.

Ventilation Shutdown Plus CO_2_ (VSD + CO_2_) is considered by the US Department of Agriculture (USDA) to be “a theoretical but not yet practical option” for depopulation [48]. It involves introducing CO_2_ after the ventilation system is shut down [49,50,51], the same process used in whole house gassing (WHG) with CO_2_ (a method which has been the subject of more research than VSD + CO_2_ and is discussed separately in the *Guidelines* and in Table A1). Because introducing large amounts of CO_2_ into a building may cause a large drop in temperature [52,53,54], VSD + CO_2_ would not be expected to kill via heatstroke, by rather by hypercapnia/hypoxia [49].

Though VSD and VSD + Heat (VSD + H) are sometimes described as killing via suffocation because the ventilation system is shut down, research shows that oxygen levels never decrease and CO_2_ levels never increase to lethal levels [50,51,55]. Post-mortem findings confirm death occurs due to hyperthermia (heatstroke) [1,55]. Different protocols have been described for use of heatstroke-based methods to kill pigs and poultry. The poultry protocol involves raising the temperature of the poultry house to 40 °C (104 °F) or higher as quickly as possible and preferably within 30 min, then maintaining a temperature of between 40 and 43.3 °C (104–110 °F) for a minimum of three hours, with a goal of 100% mortality in “as short a time as possible” [18,56]. Under experimental conditions, VSD + H caused 100% mortality of laying hens in two hours [49,50], however, when applied under commercial conditions, the VSD + H protocol required 4.5 h until no chickens (layer breeders) were observed standing [55]. In turkeys, research shows it takes 50% longer for birds to die of VSD + H compared to laying hens [51]. Although the *Guidelines* specify that VSD+ must be “applied in a manner that will produce a 100% mortality rate”, state records indicate that, in practice, VSD+H may be carried out for over 8 h and still achieve less than 100% mortality [57,58].

The *Guidelines* do not describe a specific protocol for using VSD + H to kill pigs, but recommend it only be used in facilities where enough heat can be generated to kill 95% of pigs within one hour. A case report published in the Journal of the AVMA (*JAVMA*) states that initial trials with heat alone resulted in insufficient mortality, and goes on to describe an “enhanced” method dubbed “Ventilation shutdown with the addition of high temperature and humidity” (VSD + TH), in which death by heatstroke was hastened by the use of both heaters and steam generators [1].

The protocol described starts with engineering and retrofitting barns to facilitate use of VSD + TH [1]. After transporting pigs to the barns, the building and ventilation systems are sealed, and heaters are turned on. Once the barns reach 54 °C (129.2 °F), a process that took between 15 and 94 min in the study, steam generators are turned on to increase the humidity to a minimum of 90%. After this point, the protocol describes maintaining the temperature between 49 and 65 °C (120.2–149 °F). In practice, temperatures far exceeded this range, reaching as high as 76.7 °C (170.1 °F). The protocol indicates that high temperature and humidity conditions are maintained until no sounds are audible from within the barn.

While the case report’s authors assert that VSD+TH “exceeded the requirements outlined in the AVMA depopulation guidelines of a >95% mortality rate in <1 h”, this claim has been disputed in the scientific literature [17,59]. In determining the time to death, the authors counted only the time that elapsed between the introduction of steam and the point when no sounds from the pigs were audible. However, steam was introduced only when the temperature reached 54 °C (129.2 °F), which is far above what the pork industry defines as pigs’ preferred temperature range and upper critical thermal limit: 18.3–26.7 °C (65–80 °F) and 35 °C (95 °F), respectively [60]. Measured from the point at which the ventilation system was sealed and heaters were turned on, the time required until a group of pigs fell silent averaged 90.4 min for nursery piglets and 110.3 min for finishing pigs [1]. The maximum time was 110 min and 151 min, respectively, and there were documented survivors assessed as showing signs of consciousness [1]. While such time periods appear to violate the criteria laid out in the VSD+ section of the *Guidelines*, the American Association of Swine Veterinarians has adopted the convention of setting as “time zero” the timepoint at which barn temperature reaches 54.4 °C (130 °F) [61].

Importantly, relying on animal movement or noise from vocalizations as proxies for death is problematic, as it lacks sensitivity to detect subtle behaviors (e.g., gasping) which can be performed in both conscious and unconscious animals and are highly likely to occur in animals exposed to VSD+. Previous research has shown that loss of posture and cessation of vocalizations does not always coincide with physiological markers of death or even loss of electrical brain activity [62,63,64,65,66]. Thus, latencies to death based on only “time to silent” or lying down may be overly optimistic.

### 2.1. Pathophysiology of Heatstroke

Although complete necropsy findings for animals who died by VSD, VSD+H, or VSD+TH have not been published, available research indicates that death results from severe, environmental heatstroke [1,49,55]. Heat stress occurs when the ambient temperature exceeds the thermoneutral zone. Heatstroke is the most advanced form of heat stress, with elevated temperature activating inflammatory and hemostasis cascades and leading to multiorgan failure [7,67,68,69,70,71].

The clinical sequelae of heatstroke are concerning. Across species, they include distributive shock, gastrointestinal bleeding and sloughing with attendant vomiting and hemorrhagic diarrhea, abdominal organomegaly, rhabdomyolysis, acute respiratory distress syndrome, brain injury and neurological abnormalities, multiorgan dysfunction, and coagulopathies, including disseminated intravascular coagulation (DIC), frequently ending in hemorrhagic diathesis [7,67,68,69,70,72,73,74,75]. In dogs, the cause of death in fatal heat stroke is typically shock and respiratory failure due to accumulation of frothy, hemorrhagic fluid in the airways [68].

In humans, encephalopathy predominates and neurologic symptoms are seen early in the progression of heatstroke [69], which might suggest early loss of consciousness (LOC) in animals subjected to VSD + H/TH. For two reasons, this may not occur in pigs. Porcine brain tissue is less sensitive to thermal damage compared to other species, including primates [76,77]. In addition, the porcine brain is protected from increases in body temperature by a anatomic “cooling system”, the carotid rete mirabile [7,78]. Thus, in pigs, brain injury and neurologic abnormalities may occur later in the course of heatstroke, typically as a result of hypoperfusion, hypoglycemia, and/or multiorgan dysfunction, rather than early on from thermal insult [7,67]. This may also be true for chickens and turkeys, who possess a rete mirabile ophthalmicum, believed to have a similar brain-cooling function [79,80,81,82,83].

### 2.2. VSD + TH and Burn Injuries

It can be speculated that, in pigs, VSD + TH may cause severe burns prior to LOC, especially at the higher reported temperature ranges. Pigs are frequently used in burn research because of the extensive anatomical and physiological similarities between porcine and human skin [84]. Temperature conditions at the high end of the range reported for VSD + TH are similar to those known to cause second- and third-degree hot air sauna burns (HASB) and rhabdomyolysis in humans who lose consciousness or become immobile in a sauna for as little as 30 min [1,85,86,87,88]. As discussed above, when VSD+TH is performed, the temperature within the barn may be raised as high as to 76.7 °C (170.1 °F) [1], well within the range of temperatures found in saunas (70–100 °C, or 158–212 °F) [86,89,90].

The introduction of steam with VSD+TH to raise the humidity to a minimum of 90% [1], creates humidity levels similar to those of a steam room [90]. Because of the heat-carrying capacity of steam [91,92], steam rooms are typically kept at cooler temperatures than saunas, around 43.3–48.9 °C (110–120 °F), to prevent thermal discomfort [86,89,90]. Research using ex vivo porcine skin has shown that temperature and humidity conditions similar to those created during VSD + TH (70 °C or 158 °F, relative humidity of 75%) increase the permeability of the stratum corneum as much as 50 times compared to room temperature and lead to burns of the underlying dermis before damage to the epidermis is apparent [93]. Such steam burns are generally considered more severe than burns from hot dry air [94]. Both HASB and steam burns may initially present with limited visually apparent skin changes [85,93], which would make it difficult for veterinarians overseeing VSD + TH to recognize such injuries post-mortem. While researchers in the VSD + TH report indicate they took care to avoid burning pigs with steam as it was discharged from the steam generators [95], these measures may not protect against burns from hot, highly humidified air. Research on humans and dogs suggests that, at the high end of the temperature range of VSD + TH, inhalation burns may also be possible [96,97]. This risk may be heightened by the high level of humidity, which carries heat deeper into the respiratory tract [91,92,98].

### 2.3. Affective States and Animal Welfare Implications of VSD Methods

The goal of assessing animal welfare impacts at the time of animal killing is to work towards providing a “good” death, defined by the minimization of negative experiences (e.g., pain and anxiety) in the target animals, while not eliciting fear or distress in other nearby animals. To achieve this, there are two fundamental questions: (1) how long does it take for the animal to achieve LOC; and (2) what affective states are likely to be experienced until this point [40,62]? Even though a “good” death may not be an achievable goal during all depopulations, these questions are still useful in comparing the animal welfare implications of depopulation methods.

An animal’s “affective state” refers to what the animal experiences. Defined as a complex phenomenon encompassing physiological, behavioral, and cognitive properties, affective state can be considered in terms of *valence*, i.e., whether the experience is positive, neutral, or negative, and *arousal*, or how intense the experience is [99,100]. Examples of affective states are pain, pleasure, fear, hunger, nausea, and thirst [101]. Affective states are recognized as key components to assessing the relative humaneness of different killing methods [62,102,103]. Most methods of depopulation involve a sudden change in husbandry routine and environment, which are likely to cause animals some level of anxiety due to their novelty [104].

Currently, there is only one published report attempting to explore the welfare impacts of VSD/VSD+. It is a non-peer reviewed report prepared for the US Poultry and Egg Association on VSD/VSD+ [49] and was used as the basis for including VSD/VSD+ in the *Guidelines*. However, it was not carried out under commercial conditions and its findings have been heavily criticized by avian welfare experts [105]. A subsequent peer-reviewed article summarized this research study, but did not address or report animal welfare impacts [50]. Video footage recorded by researchers during the VSD + H trials on individual birds is now publicly available [106].

Non-lethal heat stress is widely acknowledged to be detrimental to animal welfare [70] and temperature-humidity conditions that are high enough to cause death also are accepted as causing severe suffering [107,108,109]. In addition to the thermal discomfort that animals undergoing VSD + H or VSD + TH experience, the pathophysiology of heatstroke suggests several potential sources of pain: sloughing of the gastrointestinal tract [110], rhabdomyolysis [111], and stretching of organ capsule fascia as occurs with acute congestion of abdominal organs [112]. Headache is reported by humans during heatstroke [71]. Additionally, hot air burns and thermal inhalation injury, as may be possible during VSD + TH, are additional causes of severe pain [113].

While protocols for use of VSD + H in poultry recommend considerably lower temperatures than VSD + TH for pigs, research suggests that at least some birds may experience pain [55,114,115]. Under commercial conditions, VSD + H increased average core body temperature to 46–47 °C (114.8–116.6 °F) and maximal surface temperature reached 48.1 °C (118.6 °F) in some individuals [55]. These temperatures exceed the thermal nociceptive thresholds in chickens [114,115].

In addition to pain, other possible negative affective states associated with heatstroke include overheating, nausea, malaise, anxiety, fear, dizziness/disorientation, helplessness, frustration, thirst, debility, and exhaustion. Negative emotional and behavioral reactions (e.g., heat distress, aggression, and frustration) may come into play early in cases of acute heat stress and pose further risks to animal welfare [116,117,118,119,120,121], while delirium and disorientation can develop as heatstroke progresses, prior to the onset of stupor and eventually LOC [7,67,116]. Respiratory distress (dyspnea), as occurs during the terminal phase of heatstroke, is typically associated with severe anxiety in conscious animals [122].

It is not known how long animals remain conscious when subjected to VSD+H or VSD+TH, particularly if they are compromised by disease, but exposure to noxious temperatures can persist for a long time: 66 min to more than 2.5 h in pigs [1] and 4.5 h to 8 h in chickens [55,57]. Thus, it seems likely that affective states are negative and prolonged prior to LOC. These negative animal welfare impacts are cited as reasons why the European Food Safety Authority (EFSA) specifically recommends against using VSD methods for depopulation of both poultry [53] and pigs [123]. Based on the likelihood of the method being “highly painful”, the EFSA advises that VSD/VSD + H “must never be used” [123]. One veterinary journal publication opined that VSD + TH is “consistent with a general understanding of animal cruelty” [14].

## 3. Use of Heatstroke-Based Depopulation Methods

### 3.1. Rationale for Use of Heatstroke-Based Methods

The rationale for current use of VSD methods centers on their minimal requirements in terms of equipment, supplies, and labor, such that depopulation can be initiated quickly regardless of level of preparedness.

In the US, discussion about the use of VSD as a depopulation method began during the 2014–2016 HPAI outbreak, when the logistical challenges associated with depopulating poultry facilities housing up to 7 million birds each were recognized [124]. Delays in completing depopulations were believed to be contributing to on-going HPAI spread, potentially via virus from infected farms being carried to distant farms by fine particulate matter [125]. A Congressional Research Report from that time indicates that the Animal and Plant Health Inspection Service (APHIS), which manages response to HPAI outbreaks, suggested VSD as a way to “humanely euthanize” flocks while avoiding delays in deploying equipment needed for other methods [126]. APHIS subsequently developed a decision tree for selecting VSD when other depopulation methods would not be available promptly and when viral amplification at the affected site would pose a “significant threat” for further transmission [18].

Under current APHIS policy, affected flocks must be depopulated within 24 to 48 h of presumptive HPAI diagnosis [46,56]. While methods classified as “preferred” in the AVMA *Guidelines* are to be considered first, APHIS reasons that the use of VSD+ “could save the lives of thousands of birds by reducing the risk of disease spread” [46]. As discussed below (Section 5.1.2), even using VSD + H, large facilities often exceed the 48 h deadline, typically by one to two weeks (see Tables S2 and S5) [4]. However, APHIS reports that, in 2022, more rapid depopulation combined with better biosecurity is believed to have decreased farm-to-farm transmission [127].

The *Guidelines* quote extensively from two USDA documents but also make a few independent ethical assessments, including noting that “the most compelling reason to use VSD when all other methods have been ruled out is that, when done properly, it provides a quicker death, hence eliminating the chance for the birds to die over a longer period of time from distressing and devastating disease” [18]. (The same statement, adapted to pigs, is the sole justification mentioned in the Swine VSD+ section of the *Guidelines*). They also cite its ability to contain pathogens within the house and to carry out depopulation with little labor or human exposure to the birds, an important consideration particularly for highly zoonotic disease.

Because HPAI can cause up to 100% mortality, often preceded by sickness that results in poor welfare [128], depopulation may be in the birds’ interests, provided that “the welfare impact of the killing method is less severe than the suffering caused by the disease” [107]. However, during the 2022 HPAI outbreak, available state records suggest that many of the birds depopulated were not infected but were killed to prevent spread of the disease [129], a consideration that affects this utilitarian calculus.

### 3.2. Rising Use of Heatstroke-Based Methods

#### 3.2.1. Pigs

A case report was published in 2021 which explained how the decision was made to use VSD + TH to kill 243,016 pigs [1]. The COVID-19 pandemic resulted in extensive outbreaks among slaughterhouse workers, causing many plants to temporarily close [1,40,41,44,130]. Simultaneously, pork demand from the food service industry decreased dramatically [40,41]. Most modern pig and poultry production systems are very vulnerable to bottlenecks, since newborn or newly hatched animals are continuously being added to the system [40,130]. Since modern pigs are genetically selected for high growth rates and are allotted limited space, overcrowding-associated aggression and difficulty accessing food and water develop rapidly when the animals are not sent to slaughter on the date scheduled [1,40,131].

The farm in the VSD+TH report attempted to mitigate the slaughterhouse bottleneck by various methods, although the option of housing pigs outdoors was “not considered viable” [1]. Once it was determined that depopulation would be performed, methods classified as “preferred”, including CO_2_ gassing, captive bolt, gunshot, and electrocution, were reportedly considered, however all were rejected due to difficulty accessing required resources [1]. Thus, the decision was made to conduct VSD+TH.

It is unknown how many pigs were depopulated nationwide due to COVID-19 supply chain disruptions, though early on in the pandemic the National Pork Producer’s Council estimated the number could reach 10 million within the first months [130]. While the USDA does not track the methods used for depopulations unrelated to animal disease outbreaks, published accounts indicate that, in addition to VSD + TH, pigs were also depopulated by CO_2_ gassing, captive bolts, gunshot, and sodium nitrite poisoning [20,45]. With the threat that African Swine Fever may spread to the US, there has been increased interest in various pig depopulation methods, with assertions that VSD + H/TH will be needed [132,133].

#### 3.2.2. Poultry

Chickens were also depopulated by unknown methods due to COVID-19-related supply chain disruptions, with nearly 2 million birds killed in Delaware and Maryland alone [42,43].

**Figure 1:**
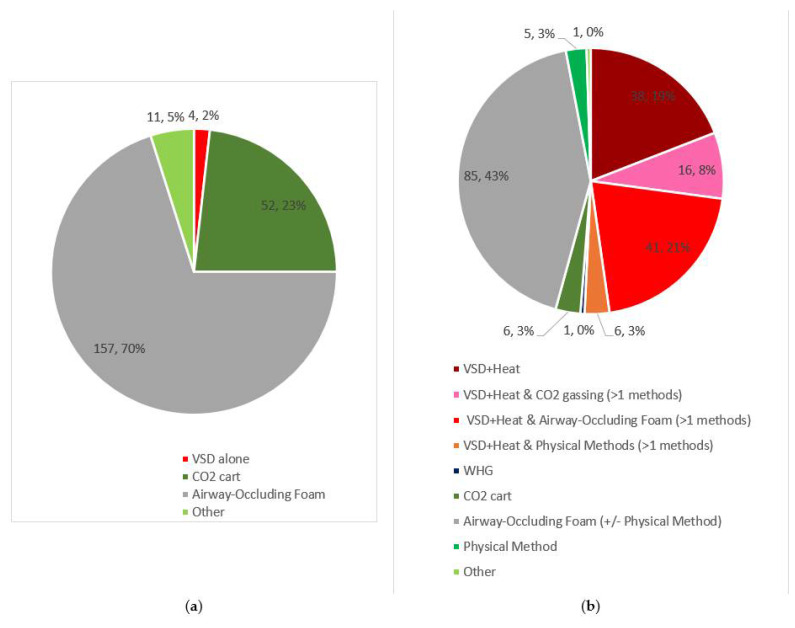
Number, percent (%) of commercial poultry depopulations related to Highly Pathogenic Avian Influenza (HPAI) by depopulation methods: (**a**), 2014–2016; (**b**) 2022. VSD = ventilation shutdown alone, VSD + Heat = ventilation shutdown + heat, CO_2_ cart = carbon dioxide gassing in cart/container, WHG = whole house gassing with CO_2_, Physical Method = captive bolt, cervical dislocation, etc.

**Figure 2:**
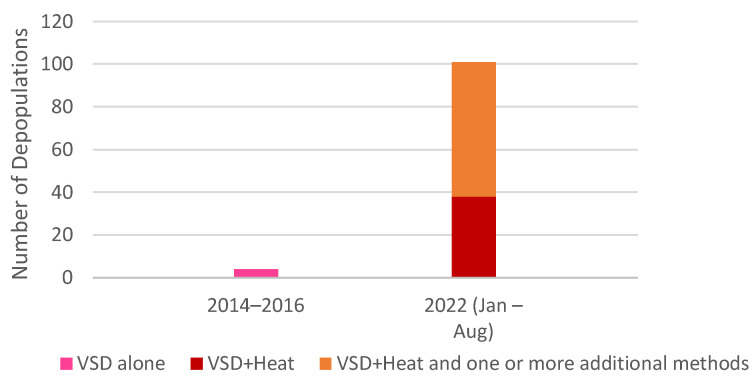
Number of commercial depopulations of poultry due to HPAI that involved the use of heatstroke-based methods.

Depopulation methods employed in response to HPAI have changed since the previous outbreak in 2014-2016 (Figure 1). VSD alone was used in only four of 224 commercial depopulations during the 2014-2016 HPAI outbreak [6], shortly after APHIS developed its “decision tree” for its use [134]. VSD was not used during numerous depopulations from 2017 to 2021 that occurred in response to various diseases [135]. However, USDA records indicate that, during the time period for which complete data is available (January–August 2022), VSD+H was used alone or in combination with other methods in over 50% of depopulations (Figure 1 and Figure 2) [3,4]. (See Supplementary Tables S1–S5 for data). 

**Figure 3:**
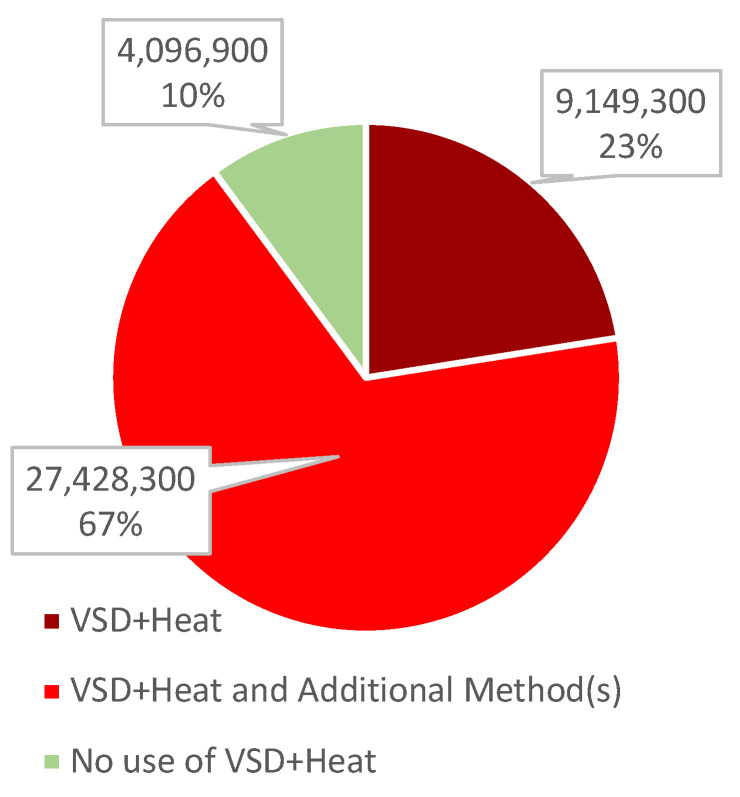
Number and percentage (%) of birds killed in HPAI depopulations in from January through August 2022.

For depopulations related to animal disease control, the USDA collects information on which method(s) are used to depopulate a given facility. However, when more than one method is used in a single facility, e.g., airway-occluding foam in some barns and VSD+H in others, the USDA reports this simply as “VSD+Heat, Foam”, without indicating the number of birds killed by each method. Therefore, the precise number of birds killed by heatstroke-based methods is unknown, ranging between 9.1 million and 36.6 million chickens, turkeys, and ducks were killed by VSD+H between January and August 2022 (Figure 3).

Despite use of VSD + H, the number of birds killed in the on-going HPAI outbreak has surpassed the total killed in the 2014–2016 outbreak [4,5]. APHIS reports that, in 2022, 85% of HPAI detections are due to wild bird introductions rather than farm-to-farm transmission [127]. Data suggest HPAI may become endemic in wild birds in the US, leading to continual, rather than sporadic, outbreaks on farms [136]. State health officials recently requested that VSD+H be classified as a “preferred” method by APHIS, which would mean other depopulation methods would not need to be ruled out in order for VSD + H to be used [137].

## 4. Other Methods of Depopulation

Use of operational slaughterhouses for production of whole carcasses or large cuts of meat requires fewer workers and has been recommended for depopulating animals in response to supply chain disruption [41]. A comprehensive discussion regarding all depopulation methods is beyond the scope of this paper. However, the Appendix A provides a detailed table (Table A1) summarizing currently available and potential depopulation methods which are scalable to large, commercial pig and poultry populations during urgent/emergent circumstances. As the table shows, numerous methods provide a more rapid loss of consciousness and less harm to animal welfare than VSD + H/TH. Several lower welfare methods, including VSD + H/TH, are considered unacceptable in the United Kingdom (UK) and European Union (EU), but are permissible in the US.

Scientific research on existing and new depopulation methods is ongoing, therefore continual review of the evidence available both, in the U.S. and globally, is necessary for the veterinary profession to remain informed, guide future research directions, and ensure that, when depopulations are performed, the most humane methods are used.

## 5. The Veterinary Profession’s Ethical Responsibilities Concerning Farm Animal Depopulations

While our focus is specifically on the US veterinary profession, any circumstance that may result in animal depopulation requires engagement by diverse stakeholders, including those who legally own the animals, animal caregivers, governmental agencies, and industry groups. Unlike the EU or UK, the US currently lacks laws regulating depopulation [138]; rather, federal and state agencies have incorporated the *Guidelines* into their policies [127,139]. Industry trade groups recognize that the US public cares about farm animal welfare [140,141,142,143] and trusts the veterinary profession to protect it [144,145]; accordingly, they may describe their depopulation methods simply as “approved by the AVMA” in media communications [146]. This effectively endows the US veterinary profession with substantial power and responsibility when it comes to animal depopulation.

The Veterinary Oath stipulates that veterinarians must balance a multitude of ethical responsibilities, including benefiting society, promoting public health, and preventing and relieving animal suffering [147]. The AVMA has affirmed that “veterinarians are, and must continually strive to be, the leading advocates for the good welfare of animals in a continually evolving society” [148]. Furthermore, the AVMA’s Animal Welfare Principles provide guidance about how animals’ lives should be ended by veterinarians: “Animals shall be treated with respect and dignity throughout their lives and, when necessary, provided a humane death” [149]. Thus, the veterinary profession must remain a strong advocate for optimizing animal welfare, even when death is brought about by depopulation.

Veterinarians frequently encounter conflicts among their obligations [150,151]. In balancing their disparate duties, the AVMA’s Principles of Veterinary Medical Ethics (PVME) recommend that, “A veterinarian should first consider the needs of the patient to prevent and relieve … suffering, … while minimizing pain or fear” [152]. However, human interests and expediency are typically prioritized both during depopulations and in the earlier phases of the disaster management cycle (e.g., prevention, preparedness) [27]. resulting in use of methods associated with poor welfare.

Depopulation-associated ethical quandaries are too vast and their causes too entrenched, for individual veterinarians to resolve on their own, particularly in moments of crisis [21,150]. As one veterinarian recounted, the enormous workload and “constant moving and acting” precluded them from having “enough time to really think about the morality” of the depopulation [21]. There is mounting evidence that depopulations, especially those involving healthy animals or associated with poor animal welfare, are potent drivers of moral distress, perpetration-induced traumatic stress, burnout, and emotional detachment in participating veterinarians [20,21,153].

All of this, along with concerns that the frequency of depopulations is likely to continue increasing [27], indicates the need for a paradigm shift regarding depopulation, to ensure that the welfare of animals is prioritized, not just *during* depopulations, but also in the planning, prevention, mitigation, and preparedness stages of disaster management [27].

We argue that the US veterinary profession has two pressing ethical duties—and opportunities—to promote this paradigm shift regarding depopulation of animals raised for food production. First, the profession should advance the most humane methods when depopulation is needed. Fulfilling this responsibility includes identifying obstacles to using higher welfare methods and working to overcome them. This is essential in maintaining the profession’s credibility as an advocate for its patients. Second, the veterinary profession should promote advancement in our scientific understanding of the causes and requirements for animal depopulations, with the goal of refining animal production systems to further minimize risks associated with the need for emergency depopulation (e.g., epidemiological studies and vaccine development).

### 5.1. Specific Opportunities for Veterinary Leadership

#### 5.1.1. Revise the AVMA Guidelines for the Depopulation of Animals

The current *Guidelines* are essential for providing information regarding depopulation methods that can be used in disaster management and for scientists seeking to improve existing methods or develop new ones. The AVMA has made positive steps in promoting animal welfare at the time of killing through the publication of the three “Humane Endings” guidance documents: (1) *Guidelines for the Euthanasia of Animals* [154]; (2) *Guidelines for the Humane Slaughter of Animals* [155], and (3) *Guidelines for the Depopulation of Animals* [18]. The AVMA’s process includes periodic revision of these guidance documents, which has so far involved numerous revisions of (1) and one (currently on-going) revision of (2), with an average of 8 years between versions. The AVMA has informally announced planned revisions of (3) ahead of its planned schedule.

The nature of the depopulation events that have occurred since the release of the *Guidelines for the Depopulation of Animals* in 2019 highlight a rapidly evolving area and suggest an urgent need for revision of the *Guidelines*. This would allow for the authors on the AVMA Panel on Depopulation to consider new evidence about the welfare impacts of depopulation methods (including those which are heatstroke-based) and facilitate open discussion about the potential contribution of the *Guidelines* to the increased use of heatstroke-based methods and how best to reverse this trend and promote practical higher welfare methods.

This could provide the Panel with the opportunity to reclassify methods associated with significant and prolonged animal suffering, such as VSD + H/TH and sodium nitrite poisoning. Options include reclassifying them as “not recommended”, creating a new “unacceptable” category to which they could be assigned, or leaving them out of the *Guidelines* altogether, as done for methods such as live burial and burning [53,123]. In addition, faster and potentially more humane depopulation methods, such as high-expansion nitrogen-filled foam (N_2_ foam), should be considered and, if deemed appropriate, added to the *Guidelines*. Development and access to higher welfare methods may allow for reclassification of some currently “preferred” methods as “permitted in constrained circumstances.”

While the AVMA does not have a direct regulatory role, the *Guidelines’* incorporation into federal and state policies [127,139] means that disease control-associated depopulations must be consistent with them if producers are to receive taxpayer-funded indemnity compensation [156]. Currently, APHIS does not offer indemnity to producers who use VSD alone, as the *Guidelines* classify it as *not recommended* [157]. Depopulation by high-expansion N_2_ foam is also ineligible for indemnification due to not being currently included in the *Guidelines* [127]. Thus, the *Guidelines* affect both the methods used during the response phase of a disaster and the financial incentive that producers have to invest in earlier phases of disaster management [27]. For example, during the preparedness phase, producers or integrators can invest in equipment, supplies, and contracts to ensure “preferred” depopulation methods are rapidly accessible in an emergency; such preparation could help prevent the occurrence of “constrained circumstances” currently needed to justify the use of heatstroke-based methods.

Reclassifying low-welfare depopulation methods would also ensure that such methods are not easily described as “AVMA-approved” in public communications. This could aid clarity and increase transparency for producers and consumers, recognized as necessary in the *Guidelines*. A basic principle of market-based economics is that consumers have knowledge of the way products are produced. In the case of animals used for food this includes knowledge of the welfare of animals during their lives on farms and at end-of-life moments, including slaughter, euthanasia, and depopulation. Such transparency helps ensure that ADF conform to consumers’ ethical standards.

#### 5.1.2. Identify Factors That Increase Animals’ Vulnerability during Emergencies

A recent essay described the need, during the recovery and reconstitution phases of animal disaster management, to “revisit the basis of animal vulnerability [to poor welfare during disasters] as a function of human design” [27].

One potential source of such vulnerability is animal housing systems. Several housing-related factors may impact how rapidly certain emergencies lead to animal welfare issues necessitating depopulation [131] and which depopulation methods are feasible [18]. For example, because poultry houses with battery cages cannot be depopulated with low- or medium-expansion water-based foam [18], viable options for rapid depopulation of large groups are currently limited to whole house gassing and VSD + H [18]. The case report about use of VSD+TH in pigs [1] points to a potential link between high stocking densities and vulnerability to depopulation in the face of supply chain disruption. According to the case report, because of concerns about increased aggression and inadequate access to food and water, “the farm realized that if packing plants closed, it would need to begin depopulating within days after the closure” due to animal welfare concerns [1]. In contrast, it was reported that farms with lower stocking densities did not experience pressure to depopulate on animal welfare grounds so rapidly [40].

In the case of poultry, a potential source of vulnerability, both to depopulation and to depopulation with low welfare methods, is the very large size of many egg production facilities, which may house 0.5 to 7 million birds at one site [124,158]. This affects the scale of depopulation, since the USDA requires all birds at the “infected premises” be killed, regardless of whether they show signs of disease. A failure in biosecurity in a single shed can lead to the depopulation of several million birds. Moreover, as APHIS has noted, the sheer number of animals at some locations makes it impossible to comply with the requirement that birds be depopulated within 48 h of a presumptive HPAI diagnosis [156,159]. Based on USDA data, all HPAI-affected farms with 1.5 million or more birds exceeded the 48 h deadline by days or weeks (Table 1 and Table S2) [3,4]. Such a delay prolongs the suffering of sick birds and increases the risk of spread to other farms and wild birds [127].

In addition, state records show that a poultry operation’s large size is offered as justification for use of VSD + H as a first-line depopulation method [57,129]. Between January and August 2022, all facilities with more than 216,000 birds used VSD + H either as their sole depopulation method or as one of multiple methods (Table S2). These 26 depopulations involved the killing of over 33 million birds (Table S2).

While the legal owners of animals, rather than individual veterinarians or the AVMA, control decisions about housing, the AVMA has policy statements on housing systems for some animals [160,161,162]. The AVMA Animal Welfare Principles acknowledge that animal housing should be “continuously evaluated, and when indicated, refined or replaced” [149]. Federal legislation that would cap the maximum number of animals per production facility has been introduced [163]. The veterinary community could consider supporting this or become involved in discussions around similar approaches.

The potential link between housing and depopulation vulnerability is but one example of how animal production as a whole should be systematically reviewed to identify potential factors that increase animals’ vulnerability to depopulation. The AVMA and others in the profession can expand existing work in emergency preparedness [164] to include more recommendations specific to animal agriculture, using as their starting point the question of “what responsible human–animal relationships should look like” during a crisis [27]. Food animal veterinarians can encourage producers, as a matter of sustainability, to publicly adopt a “cradle to grave” ethic of responsibility, including a humane death during depopulation.

#### 5.1.3. Engagement with Governmental Agencies and Legislators

Veterinarians employed by government agencies have numerous opportunities to effect change. State animal health officials, including veterinarians, decide which depopulation method(s) are used in disease-associated depopulations [127]. The contents of national and state veterinary stockpiles, as well as the training government contractors have received, may dictate which depopulation methods are feasible in the face of time constraints [37]. The veterinary profession could encourage a shift in federal policy to permit HPAI vaccination, a measure that could drastically reduce the need for depopulation [165,166,167,168,169,170].

To decrease the risk of a new HPAI outbreak, APHIS currently requires that, after depopulation, facilities meet certain biosecurity criteria before being authorized to restock with new animals [171]. In line with the AVMA PVME directive that veterinarians should “seek changes to…regulations which are contrary to the best interests of the patient” [152], veterinarians should lobby for adding a requirement that facilities demonstrate that, through management changes and/or the addition of infrastructure, they can comply with the 48 h deadline for completing depopulation without resorting to VSD + H.

The UK offers an example of how investing in research and preparedness can enable the use of more humane methods. Since 1995, the UK Department for Environmental, Food and Rural Affairs has commissioned and funded ~GBP 1.7 million in research to assess the animal welfare impacts of various depopulation methods [172]. This has resulted in the validation and use of new depopulation methods (e.g., high-expansion N_2_-filled foam and WHG) and development of an animal disease contingency plan to ensure contracts and sufficient equipment to deal with disease cases within 48 h [173]. Although the VSD protocol originated in the UK [174], it is a “last resort” and requires government authorization, which has never been granted [13,175,176].

In the US, multiple bills have recently been introduced to regulate depopulation [177,178]. If passed, they would require industrial production facilities to file comprehensive disaster mitigation plans and would deny indemnity compensation for certain depopulation methods, including VSD+. Given the large size of farms in the US, some depopulation workers have recommended developing a mobile abattoir system that would be used routinely for on-farm killing of animals (e.g., for culling sows used for breeding or “end of lay” hens) and, during emergencies, could be rapidly deployed for depopulations [179]. Increased veterinary engagement would be beneficial for the successful development of such an approach, as well as others [180].

## 6. Conclusions

The increasingly common depopulation of farm animals, especially with heatstroke-based methods, is profoundly detrimental to animal welfare and represents an urgent ethical problem for the US veterinary community. The American public cares about the treatment of animals in agriculture and relies on veterinarians to lead in matters of animal welfare. The scale of the problem necessitates that the veterinary profession champion needed change, employing the knowledge, pragmatism, creativity, and empathy that have earned veterinarians their credibility.

There is ample evidence demonstrating that heatstroke-based methods are associated with prolonged animal suffering. Furthermore, there are higher welfare alternatives being developed and currently available. The AVMA has the opportunity to change the classification of heatstroke-based methods to discourage their use and to ensure regular review and consideration of evidence-based novel depopulation methods. The reality that depopulations are no longer rare occurrences highlights the need for the profession to encourage better integration of animal welfare considerations into all aspects of animal disaster management. Finally, veterinary organizations and individual veterinarians in all segments of the profession can engage with and support legal and regulatory approaches that mitigate the risk of depopulations and the use of heatstroke-based depopulation methods.

## Figures and Tables

**Table 1:** 2022 HPAI Large Commercial Depopulations: Locations, Population Size, Time Needed to Depopulate, and Method(s) Used for the seven largest depopulations. All depopulations involved hens raised for egg production. (Time period: January–August).

Location of Farm	Number of Birds on Farm	Time Needed to Depopulate *	Method(s) Used
Iowa	5,347,500	7 days	VSD + Heat CO_2_ gassing
Iowa	5,011,700	16 days	VSD + Heat CO_2_ gassing
Wisconsin	2,750,700	16 days	VSD + Heat
Nebraska	2,118,000	18 days	VSD + Heat CO_2_ gassing
Colorado	1,936,800	17 days	VSD + Heat CO_2_ gassing
Nebraska	1,746,900	10 days	VSD + Heat CO_2_ gassing
Utah	1,501,200	18 days	VSD + Heat CO_2_ gassing

* Time is between the date HPAI diagnosis is confirmed and the date depopulation was completed. USDA does not provide the date of presumptive diagnosis.

## Data Availability

Data is contained within Supplementary Materials.

## Supplementary Materials

The following supporting information can be downloaded at: https://www.mdpi.com/article/10.3390/ani13010140/s1, Table S1. 2014-2016 HPAI Commercial Depopulation Methods, Table S2. 2022 HPAI Commercial Depopulation Methods and Duration, Table S3. USDA Records on Bird Depopulations Jan 2015 to Jan 2016, Table S4. USDA Records on Bird Depopulations Mar 2017 to Mar 2021, Table S5. USDA Records on Bird Depopulations Jun 2021 to Aug 2022.

## Appendix A

**Table A1:** Available and potential methods of depopulating pigs and poultry in the United States (US). For each method, information is provided regarding relevant literature and species, regulatory status, animal welfare considerations, feasibility, and biosecurity. Carcass disposal methods and cost associated with method are not covered. Estimated time to loss of consciousness (LOC) has been color-coded: green = near instantaneous (<1 s), yellow = 2 s to 3 min, orange = 3.1–15 min, red > 15 min. Other abbreviations: UK = United Kingdom, EU = European Union, PICC = Permitted in Constrained Circumstances, CO_2_ = carbon dioxide, N_2_ = nitrogen gas, Ar = argon.

Depopulation Method and Recommended References Containing More Detailed Information	Relevant Species	Regulatory Status [181,182,183,184,185]			Time to LOC †		Intended Mechanism of Killing	Potential Negative Affective States (Including during Handling and Restraint)	Feasibility/Practical Constraints	Biosecurity Considerations
		US (AVMA) *	UK	EU						
Gunshot (free bullet), Captive bolt [41,53,109,123,186,187,188,189,190,191]	Pigs ‡	Preferred	Allowed	Allowed			Massive diffuse brain damage, via primary injuries (e.g., direct tissue destruction, hemorrhage) and secondary injuries (e.g., cerebral edema).	Anxiety Fear Frustration Helplessness Pain Panic	Ammunition and cartridge supply and storage Interchangeable bolt heads (captive bolts) Requires animal handling/restraint Human safety considerations Specialist training Regular firearm maintenance High staffing requirement Prolonged operational time	Often results in penetrating wound to head, external loss of blood and brain tissue Close human–animal contact Moving equipment between sites
Electrocution [21,41,53,109,123,192,193,194,195,196,197,198,199,200,201,202,203,204,205,206]	Pigs	Preferred	Allowed	Allowed			Electrocution (one-step head-to-body electrocution or two step electrocution process)	Anxiety Fear Frustration Helplessness Pain Panic	Variable animal handling/restraint Human safety considerations Specialist training Specialized equipment Regular equipment maintenance Variable staffing requirements Variable operational time	With exception of electrocution trailer, close human–animal contact required Moving equipment between sites Movement of sick/injured animals
Inhaled agents (via mobile containerized gassing units, trailers or modified dumpsters) [53,107,123,207,208,209,210,211,212,213,214,215,216,217,218,219,220,221,222,223,224]	Pigs and Poultry	Preferred	Allowed (only CO_2_ for pigs)	Allowed (only CO_2_ for pigs)			Hypercapnic hypoxia/anoxia (CO_2_, CO_2_ + N_2_ or CO_2_ + Ar); Hypoxia/anoxia (N_2_ or Ar)	Anxiety Disorientation Dyspnea Fear Frustration Helplessness Nausea Pain Panic	Gas supplies Specialist training Specialist equipment Requires animal handling Human safety considerations Variable staffing requirements Prolonged operational time for the largest facilities	Close human–animal contact required Movement of animals to units Moving equipment between sites
Inhaled agents (via whole house gassing) [52,53,54,107,127,207,208,209,213,214,215,216,217,218,225,226,227,228,229,230]	Poultry	Preferred	Allowed	Allowed			Hypercapnic hypoxia	Anxiety Disorientation Dyspnea Fear Frustration Helplessness Nausea Pain Panic	Gas supplies Specialist equipment Specialist training Human safety considerations Structures may need to be modified to prevent air leakage	No live animal transport or handling required Moving equipment between sites
High-expansion § N_2_-filled foam [53,107,123,231,232,233,234,235,236,237,238,239,240]	Pigs and Poultry	Not mentioned	Allowed for poultry	Allowed for poultry			The bubbles act as a delivery mechanism for the gas, resulting in displacement of air around the animals. As the bubbles pop, the animal is exposed to 100% N_2_, resulting in death by hypoxia/anoxia.	Anxiety Disorientation Dyspnea Fear Frustration Helplessness Pain Panic	Water and foam concentrate supplies Gas supplies Specialist equipment Specialist training Minimal animal handling Human safety considerations Shed/facility infrastructure limitations on successful operation	No live animal transport or handling required Moving equipment between sites Application in whole-house, in transport vehicles or temporary penning areas
Low- or medium-expansion § foam (filled with air or inhaled agent, e.g., CO_2_, Ar, or N_2_) Includes both water-based foam and compressed air foam [53,59,107,123,231,241,242,243,244,245,246,247,248,249,250]	Pigs and Poultry	Air-filled: preferred/PICC for poultry	Not allowed	Not allowed			The foam is inhaled by the animal resulting in occlusion of airways leading to hypoxia; similar to drowning. With inhaled agent-filled foam, bubble breakdown leads to exposure to gas leading to hypoxia/anoxia +/− hypercapnia	Anxiety Disorientation Dyspnea Fear Frustration Helplessness Nausea Pain Panic	Water and foam concentrate +/− Gas supplies Specialist equipment Specialist training Minimal animal handling and/or restraint Human safety considerations Shed/facility infrastructure limits successful operation	Minimal handling required. Moving equipment between sites
Sodium Nitrite [251,252,253,254,255,256]	Pigs	PICC	Not allowed	Not allowed			Hypoxemia due to methemoglobinemia	Anxiety Debility Dyspnea (prolonged) Disorientation Fear Frustration Hunger Nausea Pain	Specific storage requirements Short expiration date Poor palatability—compounded formulation or gavaging required Pigs must be trained in advanced for voluntary ingestion Prior food deprivation required Specialist training Greater than 50% of animals have multiple bouts of vomiting Sick pigs may be inappetent, requiring gavage feeding	No live animal transport or handling required for spontaneous ingestion. Close human–animal contact required for gavage administration. Vomitus likely to be present
VSD + H VSD + TH [1,2,49,50,51,55,106,134,257]	Pigs and Poultry	PICC	Not allowed	Not allowed			Fatal heatstroke/ hyperthermia	Anxiety Debility Dyspnea Disorientation Exhaustion Fear Frustration Helplessness Nausea Malaise Overheating Pain Panic Thirst	Access to equipment (heaters, steam generators) Human safety considerations Specialist training Structure of facility may limit successful operation Retrofitting of barns required (pigs) Minimal live animal handling (poultry) Handling and transport required for pigs Variable operational time	Moving equipment between sites Transport of live pigs to retrofitted barns Vomitus, diarrhea, and respiratory secretions likely to be present
Controlled demolition [no research could be located]	Poultry	PICC	Not allowed	Not allowed			Trauma Hemorrhage Dehydration Starvation Suffocation Mechanical Asphyxia	Anxiety Chilling Disorientation Dyspnea Fear Frustration Helplessness Hunger Overheating Pain Panic Thirst	Access to demolition equipment Specialist training Cannot be used for caged poultry No live animal handling Human safety considerations High risk of <100% mortality and prolonged time to LOC for some animals Difficult/impossible to check for and euthanize survivors	No live animal transport or handling Difficulty removing carcasses Potential for scavengers to access carcasses Potential environmental contamination Moving equipment between sites

* Unlike the UK and EU, the US does not have legal or regulatory requirements about depopulation methods. When federal and state governmental agencies oversee depopulations, compliance with the AVMA *Guidelines* is often required [127,139], therefore each method’s classification in the *Guidelines* is provided. † Time to LOC is estimated based on both average time and full range from the point at which the method is initiated. ‡ While gunshot and captive bolts are recognized in the *Guidelines* as depopulation methods for poultry, we do not consider them scalable for large populations. § Expansion ratio refers to the volume of finished foam bubbles to the volume of aqueous foam solution (aqueous foam solution = foam concentrate + water). Expansion ratio affects bubble size and mechanism of killing.
