# Supplementary material for: The Rise of Heatstroke as a Method of Depopulating Pigs and Poultry: Implications for the US Veterinary Profession
Source: Animals (Basel). 2022 Dec 29;13(1):140. doi: 10.3390/ani13010140 (PMC9817707; doi:10.3390/ani13010140)
Supplement: Supplementary file 1 [file animals-13-00140-s001.zip › Supplementary Materials/Table S3. USDA Records on Bird Depopulations Jan 2015 to Jan 2016.pdf]

Premises	Incident Site	Special ID (Investigation)	Production Type (Animal Business)	Incident	Euthanasia Method	Euthanasia Completed
(b)(3) Section 1619 of the Farm bill	Iowa	Adair 01	Commercial Table Egg Layer	HPAI 2015	Foam	6/11/2015
	Wisconsin	Barron 01	Commercial Turkey Meat Bird	HPAI 2015	Foam	4/21/2015
	Wisconsin	Barron 02	Commercial Turkey Meat Bird	HPAI 2015	Foam	4/25/2015
	Wisconsin	Barron 03	Commercial Turkey Meat Bird	HPAI 2015	Foam	4/29/2015
	Wisconsin	Barron 04	Commercial Breeder Operation	HPAI 2015	Foam	5/3/2015
	Wisconsin	Barron 05	Commercial Breeder Operation	HPAI 2015	Foam	5/6/2015
	South Dakota	Beadle 01	Commercial Turkey Meat Bird	HPAI 2015	Foam	4/8/2015
	Washington	Benton 01	Backyard Producer	HPAI 2015	CO2 Gart/Container	1/5/2015
	Washington	Benton 02	Backyard Producer	HPAI 2015	CO2 Gart/Container	1/6/2015
	Minnesota	Blue Earth 01	Commercial Turkey Meat Bird	HPAI 2015	Foam	5/31/2015
	Arkansas	Boone 01	Commercial Broiler Production	HPAI 2015	Foam	3/11/2015
	Minnesota	Brown 01	Commercial Turkey Meat Bird	HPAI 2015	Foam	5/28/2015
	Minnesota	Brown 02	Commercial Breeder Operation	HPAI 2015	Captive Bolt/ TEDS	5/28/2015
	Minnesota	Brown 03	Commercial Turkey Meat Bird	HPAI 2015	Foam	5/31/2015
	Minnesota	Brown 04	Commercial Breeder Operation	HPAI 2015	Foam	6/1/2015
	Minnesota	Brown 05	Commercial Turkey Meat Bird	HPAI 2015	Foam	6/5/2015
	Iowa	Buena Vista 01	Commercial Turkey Meat Bird	HPAI 2015	Foam	4/20/2015
	Iowa	Buena Vista 02	Commercial Turkey Meat Bird	HPAI 2015	Foam	5/4/2015
	Iowa	Buena Vista 03	Commercial Turkey Meat Bird	HPAI 2015	Foam	5/5/2015
	Iowa	Buena Vista 04	Commercial Table Egg Layer	HPAI 2015	CO2 Gart/Container	5/5/2015
	Iowa	Buena Vista 05	Commercial Table Egg Layer	HPAI 2015	CO2 Gart/Container	5/28/2015
	Iowa	Buena Vista 06	Commercial Turkey Meat Bird	HPAI 2015	Foam	5/5/2015
	Iowa	Buena Vista 07	Commercial Turkey Meat Bird	HPAI 2015	Foam	5/13/2015
	Iowa	Buena Vista 08	Commercial Turkey Meat Bird	HPAI 2015	CO2 Gart/Container	5/10/2015
	Iowa	Buena Vista 09	Commercial Turkey Meat Bird	HPAI 2015	Foam	5/12/2015
	Iowa	Buena Vista 11	Commercial Turkey Meat Bird	HPAI 2015	Foam	5/14/2015
	Iowa	Buena Vista 12	Commercial Turkey Meat Bird	HPAI 2015	Foam	5/14/2015
	Iowa	Buena Vista 13	Commercial Turkey Meat Bird	HPAI 2015	Foam	5/12/2015
	Iowa	Buena Vista 14	Commercial Table Egg Pullets	HPAI 2015	CO2 Gart/Container	6/3/2015
	Iowa	Buena Vista 15	Commercial Turkey Meat Bird	HPAI 2015	Foam	5/14/2015
	Iowa	Buena Vista 16	Commercial Turkey Meat Bird	HPAI 2015	Foam	5/24/2015
	Iowa	Calhoun 01	Commercial Turkey Meat Bird	HPAI 2015	Foam	5/23/2015
	Iowa	Calhoun 02	Commercial Turkey Meat Bird	HPAI 2015	Foam	6/2/2015
	Idaho	Canyon 02	Backyard Producer	HPAI 2015	CO2 Gart/Container	1/19/2015
	Iowa	Cherokee 01	Commercial Turkey Meat Bird	HPAI 2015	Foam	5/10/2015
	Iowa	Cherokee 02	Commercial Turkey Meat Bird	HPAI 2015	Foam	5/9/2015
	Iowa	Cherokee 03	Commercial Turkey Meat Bird	HPAI 2015	Foam	5/14/2015
	Iowa	Cherokee 04	Commercial Turkey Meat Bird	HPAI 2015	Foam	5/14/2015
	Iowa	Cherokee 05	Commercial Turkey Meat Bird	HPAI 2015	Foam	5/15/2015
	Minnesota	Chippewa 01	Commercial Turkey Meat Bird	HPAI 2015	Foam	4/29/2015
	Wisconsin	Chippewa 01	Commercial Turkey Meat Bird	HPAI 2015	Foam	4/24/2015

Premises	Incident Site	Special ID (Investigation)	Production Type (Animal Business)	Incident	Euthanasia Method	Euthanasia Completed
(b)(3) Section 1619 of the Farm bill	Washington	Clallam 01	Backyard Producer	HPAI 2015	CO2 Gart/Container	1/18/2015
	Minnesota	Clay 01	Commercial Table Egg Layer	HPAI 2015	CO2 Gart/Container	5/7/2015
	Iowa	Clay 01	Commercial Table Egg Layer	HPAI 2015	CO2 Gart/Container	5/8/2015
	Iowa	Clay 02	Commercial Table Egg Pullets	HPAI 2015	CO2 Gart/Container	6/10/2015
	Minnesota	Cottonwood 01	Commercial Turkey Meat Bird	HPAI 2015	Foam	4/13/2015
	Minnesota	Cottonwood 02	Commercial Turkey Meat Bird	HPAI 2015	Foam	4/20/2015
	Oregon	Deschutes 01	Backyard Producer	HPAI 2015	CO2 Gart/Container	2/17/2015
	North Dakota	Dickey 01	Commercial Turkey Meat Bird	HPAI 2015	Foam	4/12/2015
	Nebraska	Dixon 01	Commercial Table Egg Layer	HPAI 2015	CO2 Gart/Container	6/5/2015
	Nebraska	Dixon 02	Commercial Table Egg Layer	HPAI 2015	CO2 Gart/Container	6/21/2015
	Nebraska	Dixon 03	Commercial Table Egg Pullets	HPAI 2015	CO2 Gart/Container	6/2/2015
	Nebraska	Dixon 04	Commercial Table Egg Pullets	HPAI 2015	CO2 Gart/Container	6/23/2015
	Nebraska	Dixon 05	Commercial Table Egg Pullets	HPAI 2015	CO2 Gart/Container	6/19/2015
	Nebraska	Dixon 06	Backyard Producer	HPAI 2015	Other	6/6/2015
	Oregon	Douglas 01	Backyard Producer	HPAI 2015	CO2 Gart/Container	12/21/2014
	Iowa	Hamilton 01	Commercial Turkey Meat Bird	HPAI 2015	Foam	5/28/2015
	Iowa	Hamilton 02	Commercial Turkey Meat Bird	HPAI 2015	Other	5/31/2015
	Iowa	Hamilton 03	Commercial Turkey Meat Bird	HPAI 2015	Foam	6/3/2015
	Iowa	Hamilton 04	Commercial Turkey Meat Bird	HPAI 2015	Foam	6/4/2015
	South Dakota	Hutchinson 01	Commercial Turkey Meat Bird	HPAI 2015	Foam	5/13/2015
	Missouri	Jasper 01	Commercial Turkey Meat Bird	HPAI 2015	Foam	3/12/2015
	Wisconsin	Jefferson 01	Commercial Table Egg Layer	HPAI 2015	CO2 Gart/Container	4/26/2015
	Wisconsin	Jefferson 02	Commercial Table Egg Layer	HPAI 2015	CO2 Gart/Container	5/14/2015
	Wisconsin	Jefferson 03	Commercial Table Egg Layer	HPAI 2015	CO2 Gart/Container	5/8/2015
	Montana	Judith Basin 01	Backyard Producer	HPAI 2015	Other	4/3/2015
	Wisconsin	Juneau 01	Backyard Producer	HPAI 2015	CO2 Gart/Container	4/16/2015
	Minnesota	Kandiyohi 01	Commercial Breeder Operation	HPAI 2015	Foam	4/10/2015
	Minnesota	Kandiyohi 02	Commercial Turkey Meat Bird	HPAI 2015	Foam	4/13/2015
	Minnesota	Kandiyohi 03	Commercial Breeder Operation	HPAI 2015	Foam	4/16/2015
	Minnesota	Kandiyohi 04	Commercial Breeder Operation	HPAI 2015	Foam	4/20/2015
	Minnesota	Kandiyohi 05	Commercial Turkey Meat Bird	HPAI 2015	Foam	4/23/2015
	Minnesota	Kandiyohi 06	Commercial Turkey Meat Bird	HPAI 2015	Foam	4/22/2015
	Minnesota	Kandiyohi 07	Commercial Turkey Meat Bird	HPAI 2015	Foam	4/22/2015
	Minnesota	Kandiyohi 08	Commercial Turkey Meat Bird	HPAI 2015	Foam	4/21/2015
	Minnesota	Kandiyohi 09	Commercial Turkey Meat Bird	HPAI 2015	Foam	4/24/2015
	Minnesota	Kandiyohi 10	Commercial Turkey Meat Bird	HPAI 2015	Foam	4/26/2015
	Minnesota	Kandiyohi 11	Commercial Breeder Operation	HPAI 2015	Foam	4/25/2015
	Minnesota	Kandiyohi 12	Commercial Breeder Operation	HPAI 2015	Foam	4/24/2015
	Minnesota	Kandiyohi 13	Commercial Breeder Operation	HPAI 2015	Foam	4/25/2015
	Minnesota	Kandiyohi 14	Commercial Turkey Meat Bird	HPAI 2015	Foam	4/26/2015
	Minnesota	Kandiyohi 15	Commercial Turkey Meat Bird	HPAI 2015	Foam	4/28/2015

Premises	Incident Site	Special ID (Investigation)	Production Type (Animal Business)	Incident	Euthanasia Method	Euthanasia Completed
(b)(3) Section 1619 of the Farm bill	Minnesota	Kandiyohi 16	Commercial Turkey Meat Bird	HPAI 2015	Foam	4/26/2015
	Minnesota	Kandiyohi 17	Commercial Breeder Operation	HPAI 2015	Foam	4/27/2015
	Minnesota	Kandiyohi 18	Commercial Turkey Meat Bird	HPAI 2015	Foam	4/27/2015
	Minnesota	Kandiyohi 19	Commercial Breeder Operation	HPAI 2015	Foam	4/29/2015
	Minnesota	Kandiyohi 20	Commercial Turkey Meat Bird	HPAI 2015	Foam	4/28/2015
	Minnesota	Kandiyohi 21	Commercial Turkey Meat Bird	HPAI 2015	Foam	4/27/2015
	Minnesota	Kandiyohi 22	Commercial Turkey Meat Bird	HPAI 2015	Foam	4/29/2015
	Minnesota	Kandiyohi 23	Commercial Breeder Operation	HPAI 2015	Foam	4/29/2015
	Minnesota	Kandiyohi 24	Commercial Turkey Meat Bird	HPAI 2015	Other	4/30/2015
	Minnesota	Kandiyohi 25	Commercial Breeder Operation	HPAI 2015	Captive Bolt/ TEDS	5/1/2015
	Minnesota	Kandiyohi 26	Commercial Turkey Meat Bird	HPAI 2015	Foam	5/2/2015
	Minnesota	Kandiyohi 27	Commercial Turkey Meat Bird	HPAI 2015	Foam	5/3/2015
	Minnesota	Kandiyohi 28	Commercial Turkey Meat Bird	HPAI 2015	Other	5/2/2015
	Minnesota	Kandiyohi 29	Commercial Breeder Operation	HPAI 2015	Foam	5/3/2015
	Minnesota	Kandiyohi 30	Commercial Turkey Meat Bird	HPAI 2015	Foam	5/6/2015
	Minnesota	Kandiyohi 31	Commercial Turkey Meat Bird	HPAI 2015	Foam	5/5/2015
	Minnesota	Kandiyohi 32	Commercial Turkey Meat Bird	HPAI 2015	Foam	5/9/2015
	Minnesota	Kandiyohi 33	Commercial Turkey Meat Bird	HPAI 2015	Foam	5/17/2015
	Minnesota	Kandiyohi 34	Commercial Breeder Operation	HPAI 2015	Foam	5/29/2015
	Minnesota	Kandiyohi 35	Commercial Breeder Operation	HPAI 2015	Foam	5/26/2015
	Minnesota	Kandiyohi 36	Commercial Turkey Meat Bird	HPAI 2015	Foam	5/29/2015
	Minnesota	Kandiyohi 37	Commercial Turkey Meat Bird	HPAI 2015	Foam	5/28/2015
	Minnesota	Kandiyohi 38	Commercial Turkey Meat Bird	HPAI 2015	Foam	5/28/2015
	Minnesota	Kandiyohi 39	Commercial Turkey Meat Bird	HPAI 2015	Foam	6/2/2015
	Minnesota	Kandiyohi 40	Commercial Turkey Meat Bird	HPAI 2015	Foam	6/6/2015
	California-Tulare	Kings 01	Commercial Broiler Production	HPAI 2015	Foam	2/18/2015
	South Dakota	Kingsbury 01	Commercial Turkey Meat Bird	HPAI 2015	Foam	4/10/2015
	Iowa	Kossuth 01	Commercial Breeder Operation	HPAI 2015	CO2 Gart/Container	5/8/2015
	Minnesota	Lac Qui Parle 01	Commercial Turkey Meat Bird	HPAI 2015	Foam	3/29/2015
	North Dakota	LaMoure 01	Commercial Turkey Meat Bird	HPAI 2015	Foam	4/23/2015
	Minnesota	Le Sueur 01	Commercial Turkey Meat Bird	HPAI 2015	Foam	4/14/2015
	Kansas- Basehor	Leavenworth 01	Backyard Producer	HPAI 2015	CO2 Gart/Container	3/14/2015
	Missouri	Lewis 01	Backyard Producer	HPAI 2015	Other	5/3/2015
	Minnesota	Lyon 01	Commercial Turkey Meat Bird	HPAI 2015	Foam	4/12/2015
	Iowa	Lyon 01	Commercial Table Egg Layer	HPAI 2015	CO2 Gart/Container	5/29/2015
	Iowa	Madison 01	Commercial Table Egg Layer	HPAI 2015	CO2 Gart/Container	5/16/2015
	South Dakota	McCook 01	Commercial Turkey Meat Bird	HPAI 2015	Foam	4/13/2015
	South Dakota	McPherson 01	Commercial Turkey Meat Bird	HPAI 2015	Foam	4/15/2015
	Minnesota	Meeker 01	Commercial Turkey Meat Bird	HPAI 2015	Foam	4/14/2015
	Minnesota	Meeker 02	Commercial Turkey Meat Bird	HPAI 2015	Foam	4/21/2015
	Minnesota	Meeker 03	Commercial Turkey Meat Bird	HPAI 2015	Foam	4/19/2015

Premises	Incident Site	Special ID (Investigation)	Production Type (Animal Business)	Incident	Euthanasia Method	Euthanasia Completed
(b)(3) Section 1619 of the Farm bill	Minnesota	Meeker 04	Commercial Turkey Meat Bird	HPAI 2015	Foam	4/23/2015
	Minnesota	Meeker 05	Commercial Turkey Meat Bird	HPAI 2015	Foam	4/26/2015
	Minnesota	Meeker 06	Commercial Breeder Operation	HPAI 2015	Foam	4/26/2015
	Minnesota	Meeker 07	Commercial Turkey Meat Bird	HPAI 2015	Foam	4/25/2015
	Minnesota	Meeker 08	Commercial Breeder Operation	HPAI 2015	Foam	5/2/2015
	Minnesota	Meeker 09	Commercial Turkey Meat Bird	HPAI 2015	Foam	5/16/2015
	Minnesota	Meeker 10	Commercial Breeder Operation	HPAI 2015	Foam	5/29/2015
	Missouri	Moniteau 01	Commercial Turkey Meat Bird	HPAI 2015	Foam	3/10/2015
	South Dakota	Moody 01	Commercial Table Egg Layer	HPAI 2015	CO2 Gart/Container	6/12/2015
	South Dakota	Moody 02	Commercial Turkey Meat Bird	HPAI 2015	Foam	5/30/2015
	Minnesota	Nicollet 01	Commercial Table Egg Layer	HPAI 2015	CO2 Gart/Container	5/22/2015
	Minnesota	Nobles 01	Commercial Turkey Meat Bird	HPAI 2015	Foam	4/3/2015
	Iowa	O'Brien 01	Commercial Table Egg Layer	HPAI 2015	CO2 Gart/Container	5/6/2015
	Iowa	O'Brien 02	Commercial Table Egg Layer	HPAI 2015	CO2 Gart/Container	5/2/2015
	Iowa	O'Brien 03	Backyard Producer	HPAI 2015	Other	5/9/2015
	Washington	Okanogan 01	Backyard Producer	HPAI 2015	CO2 Gart/Container	2/3/2015
	Washington	Okanogan 02	Backyard Producer	HPAI 2015	CO2 Gart/Container	2/4/2015
	Iowa	Osceola 01	Commercial Table Egg Layer	HPAI 2015	CO2 Gart/Container	5/7/2015
	Iowa	Osceola 02	Commercial Table Egg Pullets	HPAI 2015	CO2 Gart/Container	5/20/2015
	Iowa	Osceola 03	Commercial Table Egg Pullets	HPAI 2015	CO2 Gart/Container	5/15/2015
	Iowa	Osceola 04	Backyard Producer	HPAI 2015	Other	5/22/2015
	Minnesota	Otter Tail 01	Commercial Breeder Operation	HPAI 2015	Foam	4/22/2015
	Minnesota	Otter Tail 02	Commercial Turkey Meat Bird	HPAI 2015	Foam	4/24/2015
	Minnesota	Otter Tail 03	Commercial Turkey Meat Bird	HPAI 2015	Foam	5/1/2015
	Minnesota	Otter Tail 04	Commercial Breeder Operation	HPAI 2015	Foam	4/22/2015
	Iowa	Palo Alto 01	Commercial Turkey Meat Bird	HPAI 2015	Foam	5/15/2015
	Minnesota	Pipestone 01	Backyard Producer	HPAI 2015	Foam	4/20/2015
	Minnesota	Pipestone 02	Commercial Turkey Meat Bird	HPAI 2015	Foam	5/4/2015
	Iowa	Plymouth 01	Commercial Table Egg Pullets	HPAI 2015	CO2 Gart/Container	6/5/2015
	Iowa	Pocahontas 01	Commercial Turkey Meat Bird	HPAI 2015	CO2 Gart/Container	5/6/2015
	Iowa	Pocahontas 02	Commercial Turkey Meat Bird	HPAI 2015	Foam	5/12/2015
	Iowa	Pocahontas 04	Commercial Turkey Meat Bird	HPAI 2015	Foam	5/24/2015
	Minnesota	Pope 01	Commercial Breeder Operation	HPAI 2015	Foam	3/7/2015
	Minnesota	Pope 02	Commercial Breeder Operation	HPAI 2015	Foam	3/7/2015
	Minnesota	Redwood 01	Commercial Turkey Meat Bird	HPAI 2015	Foam	4/16/2015
	Minnesota	Redwood 02	Commercial Turkey Meat Bird	HPAI 2015	Foam	4/21/2015
	Minnesota	Redwood 03	Commercial Turkey Meat Bird	HPAI 2015	Foam	4/16/2015
	Minnesota	Redwood 04	Commercial Turkey Meat Bird	HPAI 2015	Foam	4/21/2015
	Minnesota	Renville 01	Commercial Turkey Meat Bird	HPAI 2015	Foam	5/4/2015
	Minnesota	Renville 02	Commercial Table Egg Layer	HPAI 2015	CO2 Gart/Container	6/1/2015
	Minnesota	Renville 03	Commercial Turkey Meat Bird	HPAI 2015	Foam	5/28/2015

Premises	Incident Site	Special ID (Investigation)	Production Type (Animal Business)	Incident	Euthanasia Method	Euthanasia Completed
(b)(3) Section 1619 of the Farm bill	Minnesota	Renville 04	Commercial Turkey Meat Bird	HPAI 2015	Foam	5/31/2015
	Minnesota	Renville 05	Commercial Breeder Operation	HPAI 2015	Foam	6/2/2015
	Minnesota	Renville 06	Commercial Turkey Meat Bird	HPAI 2015	Foam	5/30/2015
	Minnesota	Renville 07	Commercial Table Egg Pullets	HPAI 2015	CO2 Gart/Container	6/10/2015
	Minnesota	Renville 08	Commercial Turkey Meat Bird	HPAI 2015	Foam	6/4/2015
	South Dakota	Roberts 01	Commercial Turkey Meat Bird	HPAI 2015	Foam	4/16/2015
	Minnesota	Roseau 01	Commercial Turkey Meat Bird	HPAI 2015	Foam	4/20/2015
	Iowa	Sac 01	Commercial Turkey Meat Bird	HPAI 2015	Foam	4/30/2015
	Iowa	Sac 02	Commercial Turkey Meat Bird	HPAI 2015	Foam	5/10/2015
	Iowa	Sac 03	Commercial Turkey Meat Bird	HPAI 2015	Foam	5/15/2015
	Iowa	Sac 04	Commercial Turkey Meat Bird	HPAI 2015	Foam	5/20/2015
	Iowa	Sac 05	Commercial Turkey Meat Bird	HPAI 2015	Foam	5/19/2015
	Iowa	Sac 06	Commercial Turkey Meat Bird	HPAI 2015	Foam	5/22/2015
	Iowa	Sac 07	Commercial Turkey Meat Bird	HPAI 2015	Foam	5/31/2015
	Iowa	Sac 08	Commercial Turkey Meat Bird	HPAI 2015	Foam	6/5/2015
	Iowa	Sioux 01	Commercial Table Egg Layer	HPAI 2015	CO2 Gart/Container	5/12/2015
	Iowa	Sioux 02	Commercial Table Egg Layer	HPAI 2015	CO2 Gart/Container	5/20/2015
	Iowa	Sioux 03	Commercial Table Egg Layer	HPAI 2015	CO2 Gart/Container	5/13/2015
	Iowa	Sioux 04	Commercial Table Egg Layer	HPAI 2015	CO2 Gart/Container	5/11/2015
	Iowa	Sioux 05	Commercial Table Egg Pullets	HPAI 2015	CO2 Gart/Container	5/23/2015
	Iowa	Sioux 06	Commercial Table Egg Layer	HPAI 2015	CO2 Gart/Container	5/19/2015
	Iowa	Sioux 07	Commercial Table Egg Pullets	HPAI 2015	CO2 Gart/Container	5/15/2015
	Iowa	Sioux 08	Commercial Table Egg Pullets	HPAI 2015	CO2 Gart/Container	5/28/2015
	Iowa	Sioux 09	Commercial Table Egg Pullets	HPAI 2015	CO2 Gart/Container	5/16/2015
	Iowa	Sioux 10	Commercial Table Egg Layer	HPAI 2015	CO2 Gart/Container	5/29/2015
	Iowa	Sioux 13	Commercial Table Egg Layer	HPAI 2015	CO2 Gart/Container	5/23/2015
	Iowa	Sioux 14	Commercial Table Egg Pullets	HPAI 2015	CO2 Gart/Container	5/29/2015
	Iowa	Sioux 15	Backyard Producer	HPAI 2015	Other	6/3/2015
	Iowa	Sioux 16	Backyard Producer	HPAI 2015	Other	6/4/2015
	Iowa	Sioux 17	Backyard Producer	HPAI 2015	CO2 Gart/Container	5/30/2015
	Iowa	Sioux 18	Commercial Table Egg Pullets	HPAI 2015	CO2 Gart/Container	5/31/2015
	Iowa	Sioux 19	Commercial Table Egg Layer	HPAI 2015	CO2 Gart/Container	6/1/2015
	Iowa	Sioux 20	Mail Order Hatchery	HPAI 2015	CO2 Gart/Container	6/12/2015
	South Dakota	Spink 01	Commercial Turkey Meat Bird	HPAI 2015	Foam	4/21/2015
	California	Stanislaus 01	Commercial Turkey Meat Bird	HPAI 2015	Other	1/30/2015
	Minnesota	Stearns 01	Commercial Turkey Meat Bird	HPAI 2015	Foam	3/30/2015
	Minnesota	Stearns 02	Commercial Turkey Meat Bird	HPAI 2015	Foam	4/5/2015
	Minnesota	Stearns 03	Commercial Turkey Meat Bird	HPAI 2015	Foam	4/8/2015
	Minnesota	Stearns 04	Commercial Turkey Meat Bird	HPAI 2015	Foam	4/14/2015
	Minnesota	Stearns 05	Commercial Turkey Meat Bird	HPAI 2015	Foam	4/15/2015
	Minnesota	Stearns 06	Commercial Turkey Meat Bird	HPAI 2015	Foam	4/19/2015

(b)(3) Section 1619 of the Farm bill

Premises	Incident Site	Special ID (Investigation)	Production Type (Animal Business)	Incident	Euthanasia Method	Euthanasia Completed
	Minnesota	Stearns 07	Commercial Turkey Meat Bird	HPAI 2015	Foam	4/26/2015
	Minnesota	Stearns 08	Commercial Turkey Meat Bird	HPAI 2015	Foam	4/24/2015
	Minnesota	Stearns 09	Commercial Turkey Meat Bird	HPAI 2015	Foam	4/26/2015
	Minnesota	Stearns 10	Commercial Turkey Meat Bird	HPAI 2015	Foam	4/27/2015
	Minnesota	Stearns 11	Commercial Breeder Operation	HPAI 2015	Foam	4/27/2015
	Minnesota	Stearns 12	Commercial Table Egg Layer	HPAI 2015	CO2 Gart/Container	5/11/2015
	Minnesota	Stearns 13	Commercial Breeder Operation	HPAI 2015	Foam	4/27/2015
	Minnesota	Stearns 14	Commercial Turkey Meat Bird	HPAI 2015	Foam	5/1/2015
	Minnesota	Steele 01	Commercial Turkey Meat Bird	HPAI 2015	Foam	4/29/2015
	Minnesota	Swift 01	Commercial Turkey Meat Bird	HPAI 2015	Foam	4/16/2015
	Minnesota	Swift 02	Commercial Turkey Meat Bird	HPAI 2015	Foam	4/19/2015
	Minnesota	Swift 03	Commercial Turkey Meat Bird	HPAI 2015	Foam	4/29/2015
	Minnesota	Swift 04	Commercial Turkey Meat Bird	HPAI 2015	Captive Bolt/ TEDS	5/4/2015
	Minnesota	Swift 05	Commercial Turkey Meat Bird	HPAI 2015	Foam	5/5/2015
	Minnesota	Swift 06	Commercial Turkey Meat Bird	HPAI 2015	Foam	5/9/2015
	Minnesota	Swift 07	Commercial Turkey Meat Bird	HPAI 2015	Foam	5/12/2015
	Minnesota	Wadena 01	Commercial Turkey Meat Bird	HPAI 2015	Foam	4/25/2015
	Minnesota	Watonwan 01	Commercial Turkey Meat Bird	HPAI 2015	Foam	4/12/2015
	Iowa	Webster 01	Commercial Table Egg Layer	HPAI 2015	CO2 Gart/Container	6/6/2015
	Indiana	Whitley 01	Backyard Producer	HPAI 2015	CO2 Gart/Container	5/9/2015
	Iowa	Wright 01	Commercial Table Egg Layer	HPAI 2015	CO2 Gart/Container	6/12/2015
	Iowa	Wright 02	Commercial Table Egg Layer	HPAI 2015	CO2 Gart/Container	6/2/2015
	Iowa	Wright 03	Commercial Table Egg Pullets	HPAI 2015	CO2 Gart/Container	6/6/2015
	Iowa	Wright 04	Commercial Table Egg Layer	HPAI 2015	CO2 Gart/Container	5/31/2015
	Iowa	Wright 05	Commercial Table Egg Pullets	HPAI 2015	CO2 Gart/Container	6/12/2015
	Iowa	Wright 06	Commercial Table Egg Layer	HPAI 2015	CO2 Gart/Container	6/20/2015
	South Dakota	Yankton 01	Commercial Turkey Meat Bird	HPAI 2015	Foam	5/13/2015
	Indiana	Dubois 01	Commercial Turkey Meat Bird	HPAI 2016	Foam	1/16/2016
	Indiana	Dubois 01a	Commercial Turkey Meat Bird	HPAI 2016	Foam	1/15/2016
	Indiana	Dubois 02	Commercial Turkey Meat Bird	HPAI 2016	Ventilation Shutdown	1/17/2016
	Indiana	Dubois 03	Commercial Turkey Meat Bird	HPAI 2016	Foam	1/20/2016
	Indiana	Dubois 04	Commercial Turkey Meat Bird	HPAI 2016	Ventilation Shutdown	1/16/2016
	Indiana	Dubois 05	Commercial Turkey Meat Bird	HPAI 2016	Foam	1/16/2016
	Indiana	Dubois 05a	Commercial Table Egg Layer	HPAI 2016	Ventilation Shutdown	1/19/2016
	Indiana	Dubois 06	Commercial Turkey Meat Bird	HPAI 2016	Ventilation Shutdown	1/16/2016
	Indiana	Dubois 07	Commercial Turkey Meat Bird	HPAI 2016	Other	1/20/2016
	Indiana	Dubois 08	Commercial Turkey Meat Bird	HPAI 2016	Captive Bolt/ TEDS	1/19/2016
	Indiana	Dubois 09	Commercial Turkey Meat Bird	HPAI 2016	Other	1/17/2016
	Indiana	Dubois 10	Commercial Turkey Meat Bird	HPAI 2016	Other	1/18/2016
