# Supplementary material for: The Rise of Heatstroke as a Method of Depopulating Pigs and Poultry: Implications for the US Veterinary Profession
Source: Animals (Basel). 2022 Dec 29;13(1):140. doi: 10.3390/ani13010140 (PMC9817707; doi:10.3390/ani13010140)
Supplement: Supplementary file 1 [file animals-13-00140-s001.zip › Supplementary Materials/Table S4. USDA Records on Bird Depopulations Mar 2017 to Mar 2021.pdf]

Incident	Incident Site	County (Premises)	Premises	Indemnity Request Requested	Euth Complete	Euthanasia Method	Euthanasia Party	Disp Complete	Disposal Method	Disinfect Complete	Control Zone Open	Control Zone Close	Quarantine Release	Restock Approved?
LPAI 2017	Alabama	CULLMAN	(b)(3)	n/a	3/23/2017	Foam	Company	3/25/2017	Burial	pending	n/a	n/a	No	
LPAI 2017	Alabama	JACKSON	(b)(3)	n/a	3/16/2017	Cervical dislocation	State/Federal Personnel	3/16/2017	Burial	pending	n/a	n/a	4/7/2017	
LPAI 2017	Alabama	LAUDERDALE	(b)(3) Section 1619 of the Farm bill	n/a	3/10/2017	Cervical dislocation	Company	3/11/2017	Burial	3/22/2017	n/a	n/a	4/6/2017	Restock Approved
HPAI 2017	Alabama	MADISON		n/a	3/11/2017	Cervical dislocation	State/Federal Personnel	3/11/2017	Burial	pending	n/a	n/a	No	
LPAI 2017	Alabama	MADISON		n/a	3/21/2017	Cervical dislocation	State/Federal Personnel	3/21/2017	Burial	3/21/2017	n/a	n/a	No	
LPAI 2017	Alabama	PICKENS		n/a	3/21/2017	Foam and CO_2	Company	3/22/2017	Burial	pending	n/a	n/a	No	
LPAI 2017	Georgia	CHATTOOGA		n/a	3/24/2017	Foam	State/Federal Personnel	3/25/2017	Burial	pending	n/a	n/a	No	
LPAI 2017	Kentucky	CHRISTIAN		n/a	3/23/2017	Cervical dislocation	Producer	3/23/2017	Burial	pending	n/a	n/a	No	
LPAI 2017	Kentucky	CHRISTIAN		n/a	3/17/2017	Foam	Company	3/18/2017	Burial	pending	n/a	n/a	No	
LPAI 2017	Tennessee	GILES		n/a	3/6/2017	KEDS	Company	3/6/2017	Burial	4/3/2017	n/a	n/a	4/10/2017	
**HPAI 2017**	**Tennessee**	**LINCOLN**	(b)(3) Section 1619 of the Farm bill	**3/4/2017**	**3/5/2017**	**Foam**	**Multiple Parties**	**3/8/2017**	**Burial**	**No**	**3/4/2017**	**4/11/2017**	**No**	
HPAI 2017	Tennessee	LINCOLN		n/a	n/a	n/a	n/a	n/a	n/a	n/a	n/a	n/a	4/11/2017	
**HPAI 2017**	**Tennessee**	**LINCOLN**		**3/15/2017**	**3/17/2017**	**Foam**	**Contractor**	**3/17/2017**	**Burial**	**No**	**3/14/2017**	**4/11/2017**	**No**	
HPAI 2017	Tennessee	LINCOLN		n/a	n/a	n/a	n/a	n/a	n/a	n/a	n/a	n/a	4/11/2017	
AI WI LP 0317	Wisconsin	BARRON		n/a	n/a	n/a	n/a	n/a	Controlled Market	n/a	n/a	n/a	4/5/2017	

Incident	Incident Site	Start Date (Ir	Special ID	Species	Production Type	Euth Comple	Euthanasia Method	Other
HPAI 2017	Tennessee	3/3/2017	none	Chicken	Commercial Broiler Breeder	3/5/2017	Foam	
LPAI 2017	Tennessee	3/9/2017	none	Chicken	Commercial Broiler Breeder	3/6/2017	KEDS	
LPAI 2017	Alabama	3/11/2017	none	Chicken	Commercial Broiler Breeder	3/10/2017	Other	Cervical Dislocation
HPAI 2017	Alabama	3/11/2017	none	Poultry	Backyard Producer	3/11/2017	Other	Cervical Dislocation
LPAI 2018	Missouri	3/2/2018	none	Turkey	Commercial Turkey Meat Bird	3/23/2018	Humane/Controlled Slaughter	
HPAI 2017	Tennessee	3/5/2017	none	Chicken	Commercial Broiler Breeder	3/17/2017	Foam	
LPAI 2018	Texas	3/6/2018	none	Chicken	Commercial Broiler Breeder	3/8/2018	Foam	
LPAI 2017	Alabama	3/15/2017	none	Chicken	Commercial Broiler Breeder	3/21/2017	Combination-see comments	Foam / CO2 Gas
LPAI 2017	Kentucky	3/16/2017	none	Chicken	Commercial Broiler Breeder	3/17/2017	Foam	
LPAI 2017	Alabama	3/18/2017	none	Chicken	Commercial Broiler Breeder	3/23/2017	Foam	
LPAI 2017	Kentucky	3/17/2017	none	Chicken	Backyard Producer	3/23/2017	Other	Cervical Dislocation
AI WI LP 0317	Wisconsin	3/3/2017	none	Turkey	Commercial Turkey Meat Bird	4/14/2017	Humane/Controlled Slaughter	
LPAI 2017	Georgia	3/24/2017	none	Chicken	Commercial Broiler Breeder	3/24/2017	Foam	
LPAI 2018	California	10/4/2018	Stanislaus 05	Turkey	Commercial Turkey Meat Bird	11/14/2018	Humane/Controlled Slaughter	
LPAI 2019	Minnesota	10/19/2018	Kandiyohi 01	Turkey	Commercial Turkey Meat Bird	11/30/2018	Humane/Controlled Slaughter	
LPAI 2019	Minnesota	10/30/2018	Stearns 01	Turkey	Commercial Turkey Meat Bird	11/16/2018	Humane/Controlled Slaughter	
LPAI 2019	Minnesota	11/1/2018	Stearns 02	Turkey	Commercial Turkey Meat Bird	11/15/2018	Humane/Controlled Slaughter	
LPAI 2019	Minnesota	10/20/2018	Kandiyohi 02	Turkey	Commercial Turkey Meat Bird	12/7/2018	Humane/Controlled Slaughter	
LPAI 2018	California	9/4/2018	Stanislaus 01	Turkey	Commercial Turkey Meat Bird	9/8/2018	Humane/Controlled Slaughter	
LPAI 2019	Minnesota	11/1/2018	Stearns 03	Turkey	Commercial Turkey Meat Bird	12/7/2018	Humane/Controlled Slaughter	
LPAI 2018	California	9/6/2018	Stanislaus 02	Turkey	Commercial Turkey Meat Bird	9/21/2018	CO2 Whole House	
LPAI 2019	Minnesota	11/2/2018	Stearns 04	Turkey	Commercial Turkey Meat Bird	12/7/2018	Humane/Controlled Slaughter	
LPAI 2018	California	9/6/2018	Stanislaus 03	Poultry	Other	10/5/2018	CO2 Cart/Container	
LPAI 2018	California	9/6/2018	Stanislaus 04	Turkey	Commercial Turkey Meat Bird	11/6/2018	Humane/Controlled Slaughter	
LPAI 2019	Minnesota	10/22/2018	Kandiyohi 03	Turkey	Commercial Turkey Meat Bird	12/28/2018	Humane/Controlled Slaughter	
LPAI 2019	Minnesota	10/22/2018	Kandiyohi 04	Turkey	Commercial Turkey Meat Bird	1/11/2019	Humane/Controlled Slaughter	
LPAI 2019	Minnesota	1/25/2019	Chippewa 01	Turkey	Commercial Turkey Meat Bird	2/8/2019	Humane/Controlled Slaughter	
LPAI 2019	California	4/16/2019	Monterey 01	Duck	Commercial Duck Breeder	4/25/2019	CO2 Cart/Container	
LPAI 2019	California	6/25/2019	Merced 01	Duck	Backyard Producer	8/14/2019	Humane/Controlled Slaughter	

Incident Site	Special ID	Production Type	Euthanasia Method	Incident	Euth Complete
California	Riverside 285	Commercial Table Egg Pullets	CO2 Cart/Container	CA VND 2018	12/16/2018 14:00
California	Riverside 351	Commercial Table Egg Layer	CO2 Whole House	CA VND 2018	1/19/2019 19:00
California	Riverside 372	Commercial Table Egg Layer	CO2 Cart/Container	CA VND 2018	1/21/2019 19:00
California	Riverside 455	Commercial Table Egg Layer	CO2 Cart/Container	CA VND 2018	

2019-2020 HPAI events

NC- SC Combined Incident H7N3 HPAI and H7N3 LPAI (Commercial Turkey Flocks),

March-April 2020

Depopulation and Disposal

Disease	Incident Site	Special ID	Euthanasia Method	# Euth to Date	Euth Complete	Euthanasia Party
LPAI	**North Carolina**	Anson 01	Foam	16,372	3/16/2020	State Personnel
LPAI	**North Carolina**	Anson 02	Foam	24,068	3/16/2020	State Personnel
LPAI	**North Carolina**	Union 01	Foam	12,900	3/15/2020	State Personnel
LPAI	**North Carolina**	Union 02	Other	65,071	3/15/2020	Company
LPAI	**North Carolina**	Union 03	Foam	7091	3/15/2020	State Personnel
LPAI	**North Carolina**	Union 04	Foam	17,877	3/15/2020	State Personnel
LPAI	**North Carolina**	Union 05	Foam	16,974	3/16/2020	State Personnel
LPAI	**North Carolina**	Union 06	Foam	15,590	3/18/2020	State Personnel
LPAI	**North Carolina**	Union 07	Foam	20,070	3/18/2020	State Personnel
LPAI	**North Carolina**	Union 08	Combination	45,974	3/22/2020	Company
LPAI	**North Carolina**	Union 09	Foam	26,149	4/2/2020	State Personnel
LPAI	**South Carolina**	Chesterfield 01	Foam	36,649	3/15/2020	State Personnel
HPAI	**South Carolina**	Chesterfield 02	Foam	32,577	4/8/2020	State Personnel

Incident	Incident Site	Special ID	Production Type	Euthanasia Method	Euth Complete
LPAI 2021	California	NorCal 001	Live Bird Sales / Non-Slaughter	CO2 Cart/Container	3/27/2021
LPAI 2021	California	NorCal 002	Backyard Producer	CO2 Cart/Container	3/28/2021
LPAI 2021	Missouri	Webster 01	Backyard Producer	CO2 Cart/Container	11/11/2020
