# Supplementary material for: The Rise of Heatstroke as a Method of Depopulating Pigs and Poultry: Implications for the US Veterinary Profession
Source: Animals (Basel). 2022 Dec 29;13(1):140. doi: 10.3390/ani13010140 (PMC9817707; doi:10.3390/ani13010140)
Supplement: Supplementary file 1 [file animals-13-00140-s001.zip › Supplementary Materials/Table S5. USDA Records on Bird Depopulations Jun 2021 to Aug 2022.pdf]

Premises	Incident Site	Special ID	Production Type	Incident	Euthanasia Method	Euth Complete
b6	New York	Ulster 01	Backyard Producer	HPAI 2022	KEDS	2/28/2022
	New York	Dutchess 01	Backyard Producer	HPAI 2022	Cervical Dislocation	2/26/2022
	Michigan	Kalamazoo 01	Backyard Producer	HPAI 2022	CO2 Cart/Container	2/24/2022
	Maine	Knox 02	Backyard Producer	HPAI 2022	CO2 Cart/Container	2/23/2022
	Indiana	Dubois 03	Commercial Turkey Meat Bird	HPAI 2022	Foam	2/23/2022
	New York	Suffolk 01	Backyard Producer	HPAI 2022	Cervical Dislocation	2/20/2022
	Indiana	Greene 02	Commercial Turkey Meat Bird	HPAI 2022	Foam	2/19/2022
	Maine	Knox 01	Backyard Producer	HPAI 2022	CO2 Cart/Container	2/19/2022
	Indiana	Greene 01	Commercial Turkey Meat Bird	HPAI 2022	Foam	2/18/2022
	Kentucky	Fulton 01	Commercial Broiler Production	HPAI 2022	Ventilation Shutdown Plus/Foam/Cervical Dislocation	2/15/2022
	Indiana	Dubois 02	Commercial Turkey Meat Bird	HPAI 2022	Foam	2/15/2022
	Kentucky	Webster 01	Commercial Turkey Meat Bird	HPAI 2022	Ventilation Shutdown Plus/Foam	2/15/2022
	Virginia	Fauquier 01	Backyard Producer	HPAI 2022	CO2 Cart/Container	2/12/2022
	Indiana	Dubois 01	Commercial Turkey Meat Bird	HPAI 2022	Foam	2/9/2022
	Pennsylvania	Delaware 01	Live Bird Sales / Slaughter	LPAI 2022	Cervical Dislocation/Exsanguination	1/7/2022
	Minnesota	Kandiyohi 001	Commercial Turkey Meat Bird	LPAI 2022	Humane/Controlled Slaughter	12/11/2021
	Minnesota	Kandiyohi 002	Commercial Turkey Meat Bird	LPAI 2022	Foam	12/9/2021
	California	Stanislaus 01	Commercial Quail Producer	LPAI 2021	CO2 Cart/Container	10/13/2021
	Pennsylvania	York 01	Other	LPAI 2021	CO2 Cart/Container	6/11/2021

Incident Site	Special ID	Production Type	Incident	Euthanasia Method	Euth Complete
Delaware	New Castle 01	Commercial Table Egg Layer	HPAI 2022	VSD+ heat/ CO2	3/1/2022
Iowa	Pottawattamie 01	Backyard Producer	HPAI 2022	KEDS	3/1/2022
Connecticut	New London 01	Backyard Producer	HPAI 2022	CO2/CED	3/1/2022
Indiana	Dubois 04	Commercial Turkey Meat Bird	HPAI 2022	Foam	3/1/2022
Missouri	Bates 01	Backyard Producer	HPAI 2022	CO2 Cart/Container	3/5/2022
Missouri	Stoddard 01	Commercial Broiler Production	HPAI 2022	VSD+ heat/foam	3/5/2022
Iowa	Buena Vista 01	Commercial Turkey Meat Bird	HPAI 2022	VSD+ heat	3/7/2022
South Dakota	Charles Mix 01	Commercial Turkey Meat Bird	HPAI 2022	VSD+ heat/foam	3/7/2022
Maryland	Cecil 01	Commercial Table Egg Layer	HPAI 2022	VSD+ heat/CO2	3/8/2022
Maryland	Queen Anne's 01	Commercial Broiler Production	HPAI 2022	VSD+ heat	3/9/2022
Missouri	Jasper 01	Commercial Turkey Meat Bird	HPAI 2022	Foam	3/9/2022
Missouri	Lawrence 01	Commercial Turkey Breeder Replacement Hens	HPAI 2022	Foam	3/9/2022
Delaware	New Castle 02	Commercial Table Egg Pullets	HPAI 2022	VSD+ heat/CO2	3/11/2022
Kansas	Franklin 01	Backyard Producer	HPAI 2022	CO2 Cart/Container	3/12/2022
Illinois	McLean 01	Backyard Producer	HPAI 2022	CO2 Cart/Container	3/12/2022
Maine	Lincoln 01	Backyard Producer	HPAI 2022	CO2 Cart/Container	3/13/2022
Maryland	Cecil 02	Commercial Table Egg Layer	HPAI 2022	VSD+ heat/CO2	3/13/2022
South Dakota	Charles Mix 02	Commercial Turkey Meat Bird	HPAI 2022	VSD+ heat/foam	3/13/2022
Iowa	Taylor 01	Commercial Table Egg Layer	HPAI 2022	VSD+ heat/CO2/Cervical Dislocation	3/14/2022
Maine	York 01	Backyard Producer	HPAI 2022	CO2 Cart/Container	3/16/2022
Missouri	Ralls 01	Backyard Producer	HPAI 2022	CO2 Cart/Container	3/16/2022
Nebraska	Merrick 01	Backyard Producer	HPAI 2022	CO2/Barbiturate	3/16/2022
Delaware	Kent 01	Commercial Broiler Production	HPAI 2022	VSD+ heat/foam	3/17/2022
Kansas	Sedgwick 01	Backyard Producer	HPAI 2022	Cervical Dislocation	3/17/2022
Maine	Lincoln 02	Backyard Producer	HPAI 2022	CO2 Cart/Container	3/18/2022
New Hampshire	Rockingham 01	Backyard Producer	HPAI 2022	CO2/Cervical Dislocation	3/18/2022
Maine	York 02	Backyard Producer	HPAI 2022	CO2 Cart/Container	3/19/2022
South Dakota	Kingsbury 01	Commercial Turkey Meat Bird	HPAI 2022	VSD+ heat	3/19/2022
South Dakota	Hanson 01	Commercial Turkey Meat Bird	HPAI 2022	VSD+ heat/foam	3/19/2022
Kansas	Dickinson 01	Backyard Producer	HPAI 2022	CO2 Cart/Container	3/19/2022
Maine	Knox 03	Backyard Producer	HPAI 2022	CO2 Cart/Container	3/21/2022
Iowa	Warren 01	Backyard Producer	HPAI 2022	CO2 Cart/Container	3/21/2022
Nebraska	Butler 01	Commercial Broiler Production	HPAI 2022	VSD+ heat	3/22/2022
Maryland	Cecil 04	Commercial Table Egg Pullets	HPAI 2022	VSD+ heat/cervical dislocation	3/22/2022
South Dakota	Hutchinson 01	Commercial Turkey Meat Bird	HPAI 2022	VSD+ heat/foam	3/23/2022
Maine	Cumberland 01	Backyard Producer	HPAI 2022	CO2 Cart/Container	3/23/2022
South Dakota	Edmunds 01	Commercial Turkey Breeder Hens	HPAI 2022	VSD+ heat	3/23/2022
South Dakota	Hanson 02	Commercial Turkey Meat Bird	HPAI 2022	VSD+ heat/foam	3/24/2022
South Dakota	Beadle 02	Commercial Turkey Meat Bird	HPAI 2022	VSD+ heat	3/24/2022
South Dakota	Charles Mix 03	Commercial Turkey Meat Bird	HPAI 2022	VSD+ heat/foam	3/24/2022
Iowa	Buena Vista 02	Commercial Table Egg Layer	HPAI 2022	VSD+ heat/CO2	3/24/2022
Iowa	Buena Vista 03	Commercial Turkey Meat Bird	HPAI 2022	Foam/KEDS	3/24/2022
South Dakota	Beadle 01	Commercial Turkey Meat Bird	HPAI 2022	VSD+ heat	3/24/2022
South Dakota	Kingsbury 02	Commercial Table Egg Layer	HPAI 2022	CO2 Cart/Container	3/24/2022
Nebraska	Butler 02	Commercial Broiler Production	HPAI 2022	VSD+ heat	3/25/2022
Michigan	Macomb 01	Backyard Producer	HPAI 2022	Injectable	3/25/2022
Kansas	Mitchell 01	Backyard Producer	HPAI 2022	CO2 Cart/Container	3/26/2022
Minnesota	Mower 01	Backyard Producer	HPAI 2022	KEDS	3/26/2022
South Dakota	Jerauld 01	Commercial Turkey Meat Bird	HPAI 2022	VSD+ heat/foam	3/26/2022
South Dakota	Hutchinson 02	Commercial Turkey Meat Bird	HPAI 2022	VSD+ heat/foam	3/26/2022
Nebraska	Holt 01	Backyard Producer	HPAI 2022	Injectable/Barbiturate/cervical dislocation	3/26/2022
South Dakota	Bon Homme 01	Backyard Producer	HPAI 2022	CO2 Cart/Container	3/27/2022
Iowa	Franklin 01	Commercial Table Egg Pullets	HPAI 2022	VSD+ heat	3/27/2022
Missouri	Gentry 01	Backyard Producer	HPAI 2022	CO2 Cart/Container	3/27/2022
Minnesota	Stearns 01	Commercial Turkey Meat Bird	HPAI 2022	Foam/KEDS/captive bolt	3/27/2022
South Dakota	Clark 01	Commercial Turkey Meat Bird	HPAI 2022	VSD+ heat/foam	3/28/2022
Wyoming	Park 01	Backyard Producer	HPAI 2022	KEDS	3/28/2022
North Carolina	Johnston 01	Commercial Turkey Meat Bird	HPAI 2022	Foam	3/28/2022
Maine	Washington 01	Backyard Producer	HPAI 2022	CO2 Cart/Container	3/28/2022
South Dakota	Hutchinson 03	Commercial Turkey Meat Bird	HPAI 2022	VSD+ heat/foam	3/28/2022
Minnesota	Meeker 01	Commercial Turkey Meat Bird	HPAI 2022	VSD+ heat/foam	3/28/2022
New York	Suffolk 03	Backyard Producer	HPAI 2022	CO2 Cart/Container	3/29/2022
South Dakota	McPherson 01	Commercial Turkey Meat Bird	HPAI 2022	VSD+ heat/foam	3/29/2022
Wyoming	Johnson 01	Backyard Producer	HPAI 2022	Euthanasia	3/29/2022
Maine	Knox 04	Backyard Producer	HPAI 2022	CO2 Cart/Container	3/29/2022
Iowa	Hamilton 01	Commercial Turkey Meat Bird	HPAI 2022	Foam	3/29/2022
New York	Suffolk 02	Commercial Upland Gamebird Producer	HPAI 2022	CO2 Cart/Container	3/30/2022
New York	Suffolk 02	Commercial Upland Gamebird Producer	HPAI 2022	CO2 Cart/Container	3/30/2022
Minnesota	Kandiyohi 01	Commercial Turkey Meat Bird	HPAI 2022	VSD+ heat	3/30/2022
Ohio	Franklin 01	Backyard Producer	HPAI 2022	CO2 Cart/Container	3/30/2022
Maine	Lincoln 03	Backyard Producer	HPAI 2022	Injectable	3/30/2022
Massachusetts	Berkshire 01	Backyard Producer	HPAI 2022	CO2 Cart/Container	3/30/2022
South Dakota	Spink 01	Commercial Turkey Meat Bird	HPAI 2022	VSD+ heat/foam	3/30/2022

South Dakota	Bon Homme 02	Commercial Turkey Meat Bird	HPAI 2022	VSD+ heat/foam	3/30/2022
Iowa	Buena Vista 04	Commercial Turkey Meat Bird	HPAI 2022	VSD+ heat	3/30/2022
Wisconsin	Jefferson 01	Commercial Table Egg Layer	HPAI 2022	VSD+ heat	3/30/2022
Minnesota	Lac Qui Parle 01	Commercial Turkey Meat Bird	HPAI 2022	VSD+ heat/captive bolt	3/30/2022
Minnesota	Stearns 02	Backyard Producer	HPAI 2022	KEDS/captive bolt	3/31/2022
Minnesota	Morrison 01	Commercial Turkey Meat Bird	HPAI 2022	Foam	3/31/2022
South Dakota	Edmunds 02	Commercial Turkey Breeder Hens	HPAI 2022	VSD+ heat	3/31/2022
South Dakota	Brule 01	Commercial Turkey Meat Bird	HPAI 2022	VSD+ heat/foam	3/31/2022

Incident Site	Special ID	Production Type	Incident	Euthanasia Method	Euth Complete
New York	Monroe 01	Backyard Producer	HPAI 2022	CO2 Cart/Container	4/1/2022
Missouri	Jasper 02	Commercial Turkey Meat Bird	HPAI 2022	Foam	4/1/2022
South Dakota	McPherson 02	Commercial Turkey Breeder Hens	HPAI 2022	Foam	4/1/2022
North Carolina	Johnston 03	Commercial Turkey Meat Bird	HPAI 2022	Foam	4/1/2022
Iowa	Cherokee 01	Commercial Turkey Meat Bird	HPAI 2022	VSD+ Heat	4/1/2022
North Dakota	Dickey 01	Backyard Producer	HPAI 2022	CO2 Cart/Container	4/1/2022
Minnesota	Stearns 03	Commercial Turkey Meat Bird	HPAI 2022	VSD+ Heat/Foam	4/2/2022
North Dakota	Dickey 02	Commercial Turkey Meat Bird	HPAI 2022	CO2 Cart/Container	4/2/2022
South Dakota	Edmunds 03	Commercial Turkey Breeder Replacement Hens	HPAI 2022	VSD+ Heat & Foam	4/2/2022
Wisconsin	Rock 01	Backyard Producer	HPAI 2022	CO2 Cart/Container	4/2/2022
South Dakota	Charles Mix 04	Commercial Turkey Meat Bird	HPAI 2022	VSD+ Heat/Foam	4/2/2022
North Carolina	Johnston 02	Commercial Turkey Meat Bird	HPAI 2022	Foam	4/2/2022
Texas	Erath 01	Commercial Upland Gamebird Producer	HPAI 2022	CO2 Cart/Container	4/2/2022
North Carolina	Wayne 01	Commercial Turkey Meat Bird	HPAI 2022	Foam	4/2/2022
Iowa	Hamilton 02	Commercial Turkey Poult Supplier	HPAI 2022	VSD+ Heat	4/2/2022
Minnesota	Dodge 01	Commercial Turkey Meat Bird	HPAI 2022	Foam	4/3/2022
Minnesota	LeSueur 01	Commercial Turkey Meat Bird	HPAI 2022	Foam	4/3/2022
Iowa	Sac 01	Commercial Turkey Meat Bird	HPAI 2022	VSD+ Heat	4/3/2022
Nebraska	Scotts Bluff 01	Backyard Producer	HPAI 2022	Injectable	4/3/2022
Minnesota	Kandiyohi 02	Commercial Turkey Meat Bird	HPAI 2022	Foam	4/3/2022
Iowa	Humboldt 01	Commercial Table Egg Breeder	HPAI 2022	VSD+ Heat	4/3/2022
North Carolina	Wayne 02	Commercial Turkey Meat Bird	HPAI 2022	Foam	4/4/2022
Illinois	Carroll 01	Backyard Producer	HPAI 2022	KEDS	4/4/2022
South Dakota	Lake 01	Commercial Turkey Meat Bird	HPAI 2022	VSD+ Heat & Foam	4/4/2022
North Dakota	LaMoure 01	Commercial Turkey Meat Bird	HPAI 2022	VSD+ Heat & Foam	4/4/2022
South Dakota	Spink 02	Commercial Turkey Meat Bird	HPAI 2022	VSD+ Heat/Foam	4/4/2022
Minnesota	Kandiyohi 03	Commercial Turkey Breeder Hens	HPAI 2022	Foam/Cervical Dislocation	4/4/2022
Minnesota	Waseca 01	Commercial Turkey Meat Bird	HPAI 2022	VSD+ Heat & Cervical Dislocation	4/5/2022
Minnesota	Morrison 03	Commercial Turkey Meat Bird	HPAI 2022	Foam	4/5/2022
Minnesota	Kandiyohi 04	Commercial Turkey Breeder Hens	HPAI 2022	Foam	4/5/2022
North Dakota	LaMoure 02	Commercial Turkey Meat Bird	HPAI 2022	VSD+ Heat/Foam	4/5/2022
South Dakota	McPherson 03	Commercial Turkey Meat Bird	HPAI 2022	VSD+ Heat & Foam	4/5/2022
Wyoming	Fremont 01	Backyard Producer	HPAI 2022	CO2 Cart/Container	4/5/2022
Minnesota	Morrison 02	Commercial Turkey Meat Bird	HPAI 2022	Foam	4/5/2022
Iowa	Guthrie 01	Commercial Table Egg Layer	HPAI 2022	VSD+ heat/CO2	4/6/2022
South Dakota	Faulk 01	Commercial Turkey Meat Bird	HPAI 2022	VSD+ Heat	4/6/2022
Iowa	Hardin 01	Commercial Turkey Meat Bird	HPAI 2022	VSD+ Heat & Foam	4/6/2022
Minnesota	Morrison 04	Commercial Turkey Meat Bird	HPAI 2022	Foam	4/6/2022
Minnesota	Waseca 02	Commercial Turkey Meat Bird	HPAI 2022	Foam/Captive Bolt	4/6/2022
Maine	Waldo 01	Backyard Producer	HPAI 2022	CO2 Cart/Container	4/6/2022
South Dakota	Spink 03	Commercial Turkey Meat Bird	HPAI 2022	VSD+ Heat & Foam	4/6/2022
South Dakota	Clark 02	Commercial Turkey Meat Bird	HPAI 2022	VSD+ Heat & Foam	4/6/2022
North Dakota	Cass 01	Backyard Producer	HPAI 2022	CO2 Cart/Container	4/6/2022
North Carolina	Wayne 03	Commercial Broiler Production	HPAI 2022	Foam	4/6/2022
North Carolina	Wayne 04	Commercial Broiler Production	HPAI 2022	Foam	4/6/2022
Minnesota	Becker 01	Commercial Turkey Meat Bird	HPAI 2022	Foam	4/6/2022
Minnesota	Big Stone 01	Commercial Turkey Meat Bird	HPAI 2022	VSD+ Heat & Foam	4/6/2022
Missouri	Lawrence 02	Commercial Turkey Meat Bird	HPAI 2022	Foam	4/6/2022
South Dakota	Beadle 03	Commercial Turkey Meat Bird	HPAI 2022	VSD+ Heat & Foam	4/7/2022
New York	Orleans 01	Backyard Producer	HPAI 2022	CO2/Cervical Dislocation	4/7/2022
Wisconsin	Racine 01	Backyard Producer	HPAI 2022	KEDS	4/7/2022
North Carolina	Wayne 05	Commercial Turkey Meat Bird	HPAI 2022	Foam	4/7/2022
South Dakota	McPherson 04	Commercial Turkey Meat Bird	HPAI 2022	VSD+ Heat & Foam	4/7/2022
Missouri	Dade 01	Commercial Turkey Meat Bird	HPAI 2022	Foam	4/7/2022
South Dakota	Clark 03	Commercial Turkey Meat Bird	HPAI 2022	VSD+ Heat & Foam	4/7/2022
Wyoming	Park 02	Backyard Producer	HPAI 2022	CO2 Cart/Container	4/7/2022
Minnesota	Kandiyohi 05	Commercial Turkey Breeder Hens	HPAI 2022	Foam	4/7/2022
Minnesota	Stearns 04	Commercial Turkey Meat Bird	HPAI 2022	Foam	4/7/2022
Minnesota	Otter Tail 01	Commercial Turkey Meat Bird	HPAI 2022	Foam	4/7/2022
Minnesota	Renville 01	Commercial Turkey Meat Bird	HPAI 2022	Foam	4/8/2022
New York	Fulton 01	Backyard Producer	HPAI 2022	KEDS	4/8/2022
Colorado	Pitkin 01	Backyard Producer	HPAI 2022	Other	4/8/2022
Minnesota	LeSueur 02	Commercial Turkey Meat Bird	HPAI 2022	Foam	4/8/2022
South Dakota	Edmunds 04	Commercial Turkey Meat Bird	HPAI 2022	VSD+ Heat & Foam	4/8/2022
Indiana	Elkhart 01	Commercial Duck Breeder	HPAI 2022	KEDS	4/8/2022
Minnesota	Meeker 02	Commercial Turkey Meat Bird	HPAI 2022	VSD+ Heat	4/8/2022
Montana	Judith Basin 01	Backyard Producer	HPAI 2022	Cervical Dislocation	4/8/2022
North Dakota	Stutsman 01	Backyard Producer	HPAI 2022	CO2 Cart/Container	4/8/2022

Minnesota	Stearns 05	Commercial Turkey Meat Bird	HPAI 2022	Foam	4/8/2022
Minnesota	Swift 01	Commercial Turkey Meat Bird	HPAI 2022	Foam	4/8/2022
South Dakota	Yankton 01	Commercial Turkey Meat Bird	HPAI 2022	VSD+ Heat & Foam	4/9/2022
Montana	Cascade 01	Backyard Producer	HPAI 2022	CO2 Cart/Container	4/9/2022
Wisconsin	Barron 01	Commercial Turkey Meat Bird	HPAI 2022	Foam	4/10/2022
Minnesota	Todd 01	Commercial Turkey Meat Bird	HPAI 2022	Foam	4/10/2022
Minnesota	Morrison 05	Commercial Turkey Meat Bird	HPAI 2022	Foam	4/10/2022
Wyoming	Sheridan 01	Backyard Producer	HPAI 2022	All birds died. No depopulation.	4/11/2022
Kansas	McPherson 01	Commercial Turkey Breeder Hens	HPAI 2022	Foam	4/11/2022
Montana	Toole 01	Backyard Producer	HPAI 2022	Other	4/11/2022
Minnesota	Yellow Medicine 01	Commercial Turkey Meat Bird	HPAI 2022	VSD+ Heat	4/11/2022
Minnesota	Morrison 06	Commercial Broiler Production	HPAI 2022	VSD+ Heat	4/11/2022
Minnesota	Kandiyohi 06	Commercial Turkey Meat Bird	HPAI 2022	Foam	4/11/2022
Minnesota	Waseca 03	Commercial Turkey Meat Bird	HPAI 2022	VSD+ Heat/Cervical Dislocation	4/12/2022
Minnesota	Benton 02	Backyard Producer	HPAI 2022	KEDS	4/12/2022
Minnesota	Blue Earth 01	Commercial Turkey Meat Bird	HPAI 2022	Foam	4/12/2022
Minnesota	Benton 01	Backyard Producer	HPAI 2022	Other	4/12/2022
Michigan	Menominee 01	Backyard Producer	HPAI 2022	CO2 Cart/Container	4/12/2022
North Carolina	Wayne 06	Commercial Broiler Production	HPAI 2022	Foam	4/12/2022
North Dakota	Barnes 01	Backyard Producer	HPAI 2022	Other	4/12/2022
Indiana	Elkhart 02	Commercial Duck Meat Bird	HPAI 2022	KEDS	4/13/2022
Minnesota	Blue Earth 02	Commercial Turkey Meat Bird	HPAI 2022	Foam	4/13/2022
Michigan	Menominee 02	Backyard Producer	HPAI 2022	CO2 Cart/Container	4/13/2022
Minnesota	Meeker 03	Commercial Turkey Meat Bird	HPAI 2022	Foam	4/13/2022
Minnesota	Otter Tail 02	Commercial Turkey Meat Bird	HPAI 2022	Foam	4/13/2022
Idaho	Caribou 01	Backyard Producer	HPAI 2022	N/A; all birds died prior to depopulation	4/14/2022
Michigan	Macomb 02	Backyard Producer	HPAI 2022	CO2 Cart/Container	4/14/2022
Wisconsin	Polk 01	Commercial Turkey Meat Bird	HPAI 2022	Foam	4/14/2022
Utah	Utah 01	Backyard Producer	HPAI 2022	Injectable	4/14/2022
South Dakota	Deuel 01	Commercial Upland Gamebird Producer	HPAI 2022	Other	4/14/2022
Wisconsin	Sheboygan 01	Backyard Producer	HPAI 2022	CO2 Cart/Container	4/14/2022
Wisconsin	Columbia 01	Backyard Producer	HPAI 2022	CO2/Gunshot	4/15/2022
North Dakota	LaMoure 03	Backyard Producer	HPAI 2022	All birds died. No depopulation.	4/15/2022
Michigan	Livingston 01	Backyard Producer	HPAI 2022	CO2 Cart/Container	4/15/2022
Minnesota	Kandiyohi 07	Commercial Turkey Meat Bird	HPAI 2022	Foam	4/15/2022
Idaho	Gooding 01	Backyard Producer	HPAI 2022	CO2 Cart/Container	4/15/2022
Minnesota	Morrison 07	Commercial Table Egg Layer	HPAI 2022	VSD+ Heat/CO2	4/15/2022
North Dakota	Sheridan 01	Backyard Producer	HPAI 2022	CO2 Cart/Container	4/16/2022
Iowa	Osceola 01	Commercial Table Egg Layer	HPAI 2022	VSD+ heat/CO2	4/16/2022
Minnesota	Morrison 08	Commercial Turkey Meat Bird	HPAI 2022	Foam	4/16/2022
Minnesota	Morrison 09	Commercial Turkey Meat Bird	HPAI 2022	Foam	4/16/2022
Minnesota	Kandiyohi 08	Commercial Turkey Meat Bird	HPAI 2022	Foam/KEDS	4/16/2022
Minnesota	Meeker 04	Commercial Turkey Meat Bird	HPAI 2022	Foam/KEDS	4/16/2022
North Dakota	Stutsman 02	Backyard Producer	HPAI 2022	Producer depopulated.	4/17/2022
Pennsylvania	Lancaster 01	Commercial Table Egg Layer	HPAI 2022	VSD+ Heat	4/17/2022
Minnesota	Otter Tail 03	Commercial Turkey Meat Bird	HPAI 2022	VSD+ Heat/KEDS	4/17/2022
Minnesota	Swift 02	Commercial Turkey Meat Bird	HPAI 2022	Foam	4/17/2022
Colorado	La Plata 01	Backyard Producer	HPAI 2022	CO2 Cart/Container	4/17/2022
Michigan	Menominee 03	Backyard Producer	HPAI 2022	Injectable	4/18/2022
Minnesota	Morrison 10	Commercial Turkey Meat Bird	HPAI 2022	Foam	4/18/2022
Minnesota	Stearns 06	Commercial Turkey Meat Bird	HPAI 2022	VSD+ Heat/Captive Bolt	4/19/2022
Minnesota	Todd 02	Commercial Turkey Meat Bird	HPAI 2022	Foam	4/19/2022
Minnesota	Todd 03	Commercial Turkey Meat Bird	HPAI 2022	Foam	4/19/2022
Indiana	Elkhart 03	Commercial Duck Meat Bird	HPAI 2022	KEDS	4/19/2022
Minnesota	Stearns 07	Commercial Turkey Meat Bird	HPAI 2022	Foam	4/19/2022
Minnesota	Morrison 11	Commercial Turkey Meat Bird	HPAI 2022	Foam/KEDS	4/20/2022
Minnesota	Todd 04	Commercial Turkey Meat Bird	HPAI 2022	Foam	4/20/2022
Idaho	Gooding 02	Backyard Producer	HPAI 2022	CO2 Cart/Container	4/20/2022
Minnesota	Todd 05	Commercial Turkey Meat Bird	HPAI 2022	Foam	4/20/2022
North Dakota	Renville 01	Backyard Producer	HPAI 2022	CO2 Cart/Container	4/21/2022
Minnesota	Otter Tail 04	Commercial Turkey Meat Bird	HPAI 2022	VSD+ Heat	4/21/2022
Pennsylvania	Lancaster 04	Commercial Broiler Production	HPAI 2022	VSD+ Heat	4/21/2022
Idaho	Madison 01	Backyard Producer	HPAI 2022	CO2 Cart/Container	4/21/2022
Iowa	Bremer 01	Commercial Turkey Meat Bird	HPAI 2022	Foam	4/21/2022
North Dakota	Richland 01	Commercial Turkey Meat Bird	HPAI 2022	VSD+ Heat & Foam	4/22/2022
Minnesota	Yellow Medicine 02	Commercial Turkey Meat Bird	HPAI 2022	Foam	4/22/2022
Minnesota	Swift 03	Commercial Turkey Meat Bird	HPAI 2022	VSD+ Heat	4/22/2022

Colorado	Montrose 01	Commercial Broiler Breeder	HPAI 2022	CO2 Whole House	4/22/2022
Nebraska	Dixon 01	Commercial Table Egg Layer	HPAI 2022	VSD+ Heat/CO2	4/22/2022
Minnesota	Stearns 08	Commercial Turkey Meat Bird	HPAI 2022	Foam	4/23/2022
Pennsylvania	Lancaster 02	Commercial Table Egg Layer	HPAI 2022	VSD+ Heat	4/23/2022
Iowa	Kossuth 01	Backyard Producer	HPAI 2022	CO2 Cart/Container	4/23/2022
Minnesota	Rice 01	Backyard Producer	HPAI 2022	KEDS	4/23/2022
Pennsylvania	Lancaster 03	Commercial Table Egg Layer	HPAI 2022	VSD+ Heat	4/24/2022
Wisconsin	Polk 02	Backyard Producer	HPAI 2022	CO2 Cart/Container	4/24/2022
Michigan	Menominee 04	Backyard Producer	HPAI 2022	CO2 Cart/Container	4/25/2022
Montana	Missoula 01	Backyard Producer	HPAI 2022	Other	4/25/2022
North Dakota	Richland 02	Backyard Producer	HPAI 2022	Other	4/26/2022
Wisconsin	Barron 02	Commercial Turkey Meat Bird	HPAI 2022	Foam	4/27/2022
Michigan	Saginaw 01	Backyard Producer	HPAI 2022	CO2 Cart/Container	4/27/2022
Vermont	Caledonia 01	Backyard Producer	HPAI 2022	CO2 Cart/Container	4/27/2022
Minnesota	Swift 04	Backyard Producer	HPAI 2022	CO2 Cart/Container	4/27/2022
Montana	Glacier 01	Backyard Producer	HPAI 2022	CO2 Whole House	4/27/2022
Indiana	Johnson 01	Backyard Producer	HPAI 2022	Euthanasia/Cervical Dislocation/CO2	4/27/2022
Kansas	Republic 01	Backyard Producer	HPAI 2022	CO2 Cart/Container	4/28/2022
Minnesota	Carver 01	Backyard Producer	HPAI 2022	Cervical Dislocation/KEDS	4/28/2022
Wisconsin	Barron 03	Backyard Producer	HPAI 2022	Producer depopulated.	4/28/2022
Minnesota	Stearns 09	Commercial Turkey Breeder Hens	HPAI 2022	Foam	4/28/2022
Pennsylvania	Lancaster 06	Commercial Broiler Breeder Pullets	HPAI 2022	Foam	4/28/2022
Michigan	Wexford 01	Backyard Producer	HPAI 2022	CO2 Cart/Container	4/28/2022
Nebraska	Washington 01	Backyard Producer	HPAI 2022	Injectable barbiturate/cervical dislocation	4/28/2022
Pennsylvania	Lancaster 05	Commercial Table Egg Layer	HPAI 2022	VSD+ Heat	4/29/2022
Wisconsin	Oconto 01	Other	HPAI 2022	CO2 Cart/Container	4/29/2022
Wisconsin	Fond du Lac 01	Backyard Producer	HPAI 2022	KEDS	4/29/2022
Pennsylvania	Lancaster 07	Commercial Duck Meat Bird	HPAI 2022	VSD+ Heat	4/29/2022
Montana	Pondera 01	Backyard Producer	HPAI 2022	Cervical Dislocation	4/29/2022
Michigan	Branch 01	Backyard Producer	HPAI 2022	Injectable	4/30/2022
Minnesota	Morrison 12	Backyard Producer	HPAI 2022	CO2 Cart/Container	4/30/2022
Minnesota	Chisago 01	Backyard Producer	HPAI 2022	CO2 Cart/Container	4/30/2022
Alaska	Matanuska Susitna (	Backyard Producer	HPAI 2022	CO2 Cart/Container	4/30/2022
Montana	Fergus 01	Backyard Producer	HPAI 2022	Cervical Dislocation	4/30/2022
Oklahoma	Sequoyah 01	Commercial Broiler Breeder	HPAI 2022	Cervical Dislocation	4/30/2022
Utah	Cache 01	Commercial Table Egg Layer	HPAI 2022	VSD+ heat/CO2	5/13/2022
Nebraska	Knox 01	Commercial Table Egg Layer	HPAI 2022	VSD+ heat/CO2	5/15/2022

Premises	Incident Site	Special ID	Production Type	Incident	Euthanasia Method	Euth Complete
(b)(3) Section 1619 of the Farm Bill [redacted]	Minnesota	Chisago 02	Backyard Producer	HPAI 2022	KEDS/CO2	5/1/2022
[redacted]	Wisconsin	Barron 04	Commercial Turkey Meat Bird	HPAI 2022	VSD+ Heat	5/1/2022
[redacted]	Wisconsin	Barron 05	Commercial Turkey Meat Bird	HPAI 2022	VSD+ Heat & Foam	5/2/2022
[redacted]	South Dakota	Beadle 04	Commercial Turkey Meat Bird	HPAI 2022	VSD+ Heat & Foam	5/2/2022
[redacted]	South Dakota	Hamlin 01	Backyard Producer	HPAI 2022	N/A	5/2/2022
[redacted]	Minnesota	Chisago 03	Backyard Producer	HPAI 2022	CO2 Cart/Container	5/2/2022
[redacted]	Montana	Gallatin 01	Backyard Producer	HPAI 2022	Cervical Dislocation	5/2/2022
[redacted]	Wisconsin	Polk 03	Backyard Producer	HPAI 2022	CO2 Cart/Container	5/3/2022
[redacted]	Minnesota	Lyon 01	Commercial Turkey Meat Bird	HPAI 2022	Foam	5/3/2022
[redacted]	Wisconsin	Sauk 01	Backyard Producer	HPAI 2022	CO2 Cart/Container	5/3/2022
[redacted]	South Dakota	Day 01	Backyard Producer	HPAI 2022	Cervical Dislocation	5/3/2022
[redacted]	Iowa	Bremer 02	Backyard Producer	HPAI 2022	CO2 Cart/Container	5/3/2022
[redacted]	Illinois	Kane 01	Backyard Producer	HPAI 2022	KEDS	5/4/2022
[redacted]	Pennsylvania	Berks 02	Commercial Duck Meat Bird	HPAI 2022	VSD+ Heat & Foam	5/4/2022
[redacted]	Pennsylvania	Berks 01	Commercial Duck Breeder	HPAI 2022	Foam	5/4/2022
[redacted]	Minnesota	Carver 02	Backyard Producer	HPAI 2022	KEDS	5/4/2022
[redacted]	Montana	Fergus 02	Backyard Producer	HPAI 2022	Cervical Dislocation	5/4/2022
[redacted]	Wisconsin	Pierce 01	Backyard Producer	HPAI 2022	CO2 Cart/Container	5/5/2022
[redacted]	Minnesota	Anoka 01	Backyard Producer	HPAI 2022	CO2 Cart/Container	5/5/2022
[redacted]	Wisconsin	Barron 06	Commercial Turkey Meat Bird	HPAI 2022	VSD+ Heat	5/6/2022
[redacted]	Michigan	Oakland 01	Backyard Producer	HPAI 2022	CO2 Cart/Container	5/6/2022
[redacted]	Washington	Pacific 01	Backyard Producer	HPAI 2022	CO2 Cart/Container	5/6/2022
[redacted]	Oregon	Linn 01	Backyard Producer	HPAI 2022	CO2 Cart/Container & Captive Bolt	5/6/2022
[redacted]	Washington	Spokane 01	Backyard Producer	HPAI 2022	CO2 Cart/Container & Gunshot	5/7/2022
[redacted]	Pennsylvania	Berks 03	Commercial Poultry Slaughter	HPAI 2022	Foam	5/8/2022
[redacted]	Pennsylvania	Berks 04	Commercial Duck Breeder	HPAI 2022	Foam	5/8/2022
[redacted]	Pennsylvania	Lancaster 08	Commercial Table Egg Layer	HPAI 2022	VSD+ Heat	5/9/2022
[redacted]	Minnesota	Todd 06	Backyard Producer	HPAI 2022	CO2 Cart/Container	5/9/2022
[redacted]	Colorado	Weld 01	Commercial Table Egg Layer	HPAI 2022	CO2 & VSD+ Heat	5/9/2022
[redacted]	Minnesota	Crow Wing 01	Backyard Producer	HPAI 2022	CO2 Cart/Container	5/9/2022
[redacted]	Pennsylvania	Berks 05	Commercial Duck Meat Bird	HPAI 2022	VSD+ Heat	5/10/2022
[redacted]	Idaho	Canyon 01	Backyard Producer	HPAI 2022	CO2 Cart/Container	5/10/2022
[redacted]	Washington	Pierce 02	Backyard Producer	HPAI 2022	CO2 Cart/Container	5/10/2022
[redacted]	Michigan	Muskegon 01	Commercial Turkey Meat Bird	HPAI 2022	VSD+ Heat	5/10/2022
[redacted]	Idaho	Ada 02	Backyard Producer	HPAI 2022	CO2 Cart/Container	5/11/2022
[redacted]	Minnesota	Chisago 04	Backyard Producer	HPAI 2022	CO2 Cart/Container	5/11/2022
[redacted]	Minnesota	Chisago 05	Backyard Producer	HPAI 2022	CO2 Cart/Container	5/11/2022
[redacted]	Minnesota	Grant 01	Backyard Producer	HPAI 2022	CO2 Cart/Container	5/11/2022
[redacted]	Utah	Cache 02	Backyard Producer	HPAI 2022	CO2 Cart/Container	5/11/2022
[redacted]	Washington	Clallam 01	Backyard Producer	HPAI 2022	CO2 Cart/Container	5/12/2022
[redacted]	Washington	Clallam 02	Backyard Producer	HPAI 2022	CO2 Cart/Container	5/12/2022
[redacted]	Wisconsin	Barron 07	Commercial Turkey Meat Bird	HPAI 2022	Foam	5/12/2022
[redacted]	Wyoming	Lincoln 01	Backyard Producer	HPAI 2022	Cervical Dislocation	5/12/2022
[redacted]	Indiana	Allen 01	Backyard Producer	HPAI 2022	Injectable	5/12/2022
[redacted]	Washington	Whatcom 01	Backyard Producer	HPAI 2022	CO2 Cart/Container	5/13/2022
[redacted]	Idaho	Ada 03	Backyard Producer	HPAI 2022	CO2 Cart/Container	5/13/2022
[redacted]	Washington	Okanogan 01	Backyard Producer	HPAI 2022	CO2 Cart/Container	5/13/2022

(b)(3) Section 1619 of the Farm Bill

[redacted]

Wisconsin	Marinette 01	Backyard Producer	HPAI 2022	CO2 Cart/Container	5/14/2022
Washington	Pierce 01	Backyard Producer	HPAI 2022	CO2 Cart/Container & Gunshot	5/14/2022
Pennsylvania	Berks 06	Commercial Table Egg Breeder	HPAI 2022	VSD+ Heat	5/15/2022
Minnesota	Clay 01	Backyard Producer	HPAI 2022	N/A	5/15/2022
Wisconsin	Dunn 01	Backyard Producer	HPAI 2022	Cervical Dislocation	5/15/2022
Illinois	Boone 01	Backyard Producer	HPAI 2022	Cervical Dislocation	5/16/2022
Pennsylvania	Berks 07	Commercial Table Egg Layer	HPAI 2022	VSD+ Heat	5/16/2022
Idaho	Canyon 02	Backyard Producer	HPAI 2022	CO2 Cart/Container	5/16/2022
Idaho	Ada 04	Backyard Producer	HPAI 2022	CO2 Cart/Container & Injectable	5/17/2022
Utah	Salt Lake 01	Backyard Producer	HPAI 2022	CO2 Cart/Container	5/17/2022
North Dakota	Burke 01	Backyard Producer	HPAI 2022	CO2 Cart/Container	5/17/2022
Pennsylvania	Berks 08	Commercial Duck Breeder	HPAI 2022	Foam	5/18/2022
New Jersey	Monmouth 01	Backyard Producer	HPAI 2022	CO2 Cart/Container	5/18/2022
Minnesota	Kandiyohi 09	Commercial Turkey Breeder Toms	HPAI 2022	Foam	5/18/2022
Oregon	Lane 01	Backyard Producer	HPAI 2022	CO2 Cart/Container	5/18/2022
Colorado	Jefferson 01	Animal Rescue / Rehabilitation	HPAI 2022	Injectable	5/18/2022
Minnesota	Polk 01	Backyard Producer	HPAI 2022	CO2 Cart/Container	5/19/2022
Idaho	Canyon 05	Backyard Producer	HPAI 2022	CO2 Cart/Container	5/19/2022
Idaho	Canyon 04	Backyard Producer	HPAI 2022	CO2 Cart/Container	5/19/2022
Idaho	Canyon 07	Backyard Producer	HPAI 2022	CO2 Cart/Container	5/20/2022
Idaho	Ada 06	Backyard Producer	HPAI 2022	CO2 Cart/Container	5/20/2022
Minnesota	Dakota 01	Commercial Turkey Meat Bird	HPAI 2022	Foam & KEDS	5/20/2022
South Dakota	Codington 01	Backyard Producer	HPAI 2022	Cervical Dislocation	5/21/2022
Pennsylvania	Berks 09	Commercial Duck Breeder	HPAI 2022	Foam	5/22/2022
Idaho	Canyon 09	Backyard Producer	HPAI 2022	CO2 Cart/Container	5/23/2022
Utah	Sanpete 01	Commercial Turkey Meat Bird	LPAI 2022	Humane/Controlled Slaughter	5/23/2022
Wisconsin	Bayfield 01	Backyard Producer	HPAI 2022	CO2 Cart/Container	5/23/2022
Idaho	Ada 09	Backyard Producer	HPAI 2022	CO2 Cart/Container	5/23/2022
Washington	Thurston 01	Backyard Producer	HPAI 2022	CO2 Cart/Container	5/24/2022
Idaho	Canyon 03	Backyard Producer	HPAI 2022	CO2 Cart/Container	5/24/2022
Idaho	Ada 01	Backyard Producer	HPAI 2022	CO2 Cart/Container	5/24/2022
Idaho	Canyon 10	Backyard Producer	HPAI 2022	CO2 Cart/Container	5/24/2022
Idaho	Canyon 06	Backyard Producer	HPAI 2022	CO2 Cart/Container	5/25/2022
Washington	King 01	Backyard Producer	HPAI 2022	CO2 Cart/Container	5/26/2022
Idaho	Ada 10	Backyard Producer	HPAI 2022	CO2 Cart/Container	5/26/2022
Washington	King 02	Backyard Producer	HPAI 2022	CO2 Cart/Container	5/27/2022
Washington	King 03	Backyard Producer	HPAI 2022	CO2 Cart/Container	5/27/2022
Minnesota	Becker 02	Backyard Producer	HPAI 2022	CO2 Cart/Container	5/27/2022
Idaho	Ada 11	Backyard Producer	HPAI 2022	CO2 Cart/Container	5/27/2022
Washington	Snohomish 01	Backyard Producer	HPAI 2022	CO2 Cart/Container	5/28/2022

Premises	Incident Site	Special ID	Production Type	Incident	Euthanasia Method	Euth Complete
(b)(3) Section 1619 of the Farm Bill [redacted]	Washington	Snohomish 02	Backyard Producer	HPAI 2022	Longnetting/CO2 Cart/Container	6/1/2022
[redacted]	Pennsylvania	Berks 10	Commercial Duck Meat Bird	HPAI 2022	VSD+ Heat	6/2/2022
[redacted]	Indiana	Allen 02	Backyard Producer	HPAI 2022	Injectable	6/3/2022
[redacted]	Indiana	Allen 03	Backyard Producer	HPAI 2022	Injectable	6/3/2022
[redacted]	Washington	King 04	Backyard Producer	HPAI 2022	CO2 Cart/Container	6/6/2022
[redacted]	North Dakota	McHenry 01	Backyard Producer	HPAI 2022	Cervical Dislocation	6/6/2022
[redacted]	Washington	Snohomish 04	Backyard Producer	HPAI 2022	CO2 Cart/Container	6/8/2022
[redacted]	Washington	Snohomish 03	Backyard Producer	HPAI 2022	CO2 Cart/Container	6/8/2022
[redacted]	Oregon	Polk 01	Backyard Producer	HPAI 2022	CO2 Cart/Container	6/9/2022
[redacted]	Washington	Yakima 01	Backyard Producer	HPAI 2022	CO2 Cart/Container	6/10/2022
[redacted]	Colorado	Weld 03	Commercial Table Egg Pullets	HPAI 2022	CO2 Cart/Container	6/10/2022
[redacted]	Washington	Yakima 02	Backyard Producer	HPAI 2022	CO2 Cart/Container	6/11/2022
[redacted]	Utah	Salt Lake 02	Petting Zoo/Exhibition Farm	HPAI 2022	CO2 Cart/Container	6/11/2022
[redacted]	Wyoming	Sheridan 02	Backyard Producer	HPAI 2022	N/A	6/13/2022
[redacted]	Washington	King 05	Backyard Producer	HPAI 2022	CO2 Cart/Container	6/18/2022
[redacted]	Washington	King 06	Backyard Producer	HPAI 2022	Cervical Dislocation	6/18/2022
[redacted]	Washington	Yakima 03	Backyard Producer	HPAI 2022	CO2 Cart/Container	6/18/2022
[redacted]	Washington	Snohomish 05	Backyard Producer	HPAI 2022	Firearm/CO2 Cart/Container	6/20/2022
[redacted]	Colorado	Weld 02	Commercial Table Egg Layer	HPAI 2022	VSD+ heat/CO2	6/24/2022
[redacted]	Washington	Yakima 04	Backyard Producer	HPAI 2022	CO2 Cart/Container	6/28/2022
[redacted]	Maine	Cumberland 02	Backyard Producer	HPAI 2022	Injectable	6/29/2022
[redacted]	Washington	Kitsap 01	Backyard Producer	HPAI 2022	CO2 Cart/Container	6/30/2022

Premises	Incident Site	Special ID	Production Type	Incident	Euthanasia Method	Euth Complete
(b)(3) Section 1619 of the Farm bill [redacted]	Utah	Salt Lake 03	Backyard Producer	HPAI 2022	CO2 Cart/Container	7/1/2022
	Washington	Jefferson 01	Backyard Producer	HPAI 2022	CO2 Cart/Container	7/2/2022
	Oregon	Linn 02	Backyard Producer	HPAI 2022	CO2 Cart/Container	7/2/2022
	Nevada	Carson City 01	Backyard Producer	HPAI 2022	CO2 Cart/Container	7/8/2022
	Oregon	Deschutes 01	Backyard Producer	HPAI 2022	CO2 Cart/Container	7/11/2022
	Utah	Sanpete 01	Commercial Turkey Meat Bird	HPAI 2022	VSD+ heat	7/13/2022
	Oregon	Deschutes 02	Backyard Producer	HPAI 2022	CO2 Cart/Container	7/14/2022
	Oregon	Deschutes 03	Backyard Producer	HPAI 2022	CO2 Cart/Container	7/15/2022
	Washington	Snohomish 06	Backyard Producer	HPAI 2022	CO2 Cart/Container	7/18/2022
	Utah	Sanpete 02	Commercial Turkey Meat Bird	HPAI 2022	VSD+ heat/CO2	7/19/2022
	Oregon	Deschutes 04	Backyard Producer	HPAI 2022	CO2 Cart/Container	7/20/2022
	Florida	Seminole 01	Backyard Producer	HPAI 2022	CO2 Cart/Container	7/22/2022
	Montana	Flathead 01	Backyard Producer	HPAI 2022	Cervical Dislocation	7/23/2022
	Washington	Jefferson 02	Backyard Producer	HPAI 2022	CO2 Cart/Container	7/23/2022
	Utah	Sanpete 03	Commercial Turkey Meat Bird	HPAI 2022	VSD+ heat/CO2/Cervical Dislocation	7/26/2022
	Oregon	Coos 01	Backyard Producer	HPAI 2022	Long range shot	7/27/2022

Premises	Incident Site	Special ID	Production Type	Incident	Euthanasia Method	Euth Complete
00RM8UA	California	Sacramento 01	Backyard Producer	HPAI 2022	Other	8/9/2022
0012N0A	Florida	Osceola 01	Backyard Producer	HPAI 2022	Injectable	8/11/2022
00RM5FH	Pennsylvania	Northampton 01	Backyard Producer	HPAI 2022	CO2 Cart/Container	8/11/2022
00R0EZ6	Washington	Walla Walla 01	Backyard Producer	HPAI 2022	CO2 Cart/Container	8/16/2022
00HUWA5	Washington	Kitsap 02	Backyard Producer	HPAI 2022	CO2 Cart/Container	8/19/2022
0018RCR	California	Butte 01	Backyard Producer	HPAI 2022	CO2 Cart/Container	8/19/2022
00HTED9	California	Contra Costa 01	Backyard Producer	HPAI 2022	CO2 Cart/Container	8/22/2022
0011EDS	Utah	Weber 01	Backyard Producer	HPAI 2022	Injectable	8/24/2022
00LXN42	Washington	Pierce 04	Backyard Producer	HPAI 2022	CO2/long netting/cervical dislocation	8/25/2022
001MDJR	Washington	Cowlitz 01	Backyard Producer	HPAI 2022	CO2 Cart/Container	8/25/2022
00RLZJ6	Georgia	Henry 01	Animal Rescue / Rehabilitation	HPAI 2022	CO2 Cart/Container	8/26/2022
000N5TC	California	Fresno 01	Commercial Broiler Breeder	HPAI 2022	VSD+ Heat & Foam	8/26/2022
000N5SE	California	Fresno 02	Commercial Broiler Breeder	HPAI 2022	Foam	8/27/2022
00RLLT3	Virginia	Caroline 01	Non Animal Production	HPAI 2022	CO2 Cart/Container	8/28/2022
00RLR2X	California	Tuolumne 02	Commercial Turkey Meat Bird	HPAI 2022	Cervical Dislocation	8/28/2022
00RLRRK	California	Tuolumne 01	Commercial Turkey Meat Bird	HPAI 2022	Foam	8/29/2022
00RLRMU	California	Sacramento 02	Commercial Turkey Meat Bird	HPAI 2022	CO2 Cart/Container	8/29/2022
00RLLGT	Minnesota	Meeker 05	Commercial Turkey Meat Bird	HPAI 2022	Foam	8/29/2022
00NB9H0	California	Tuolumne 03	Commercial Turkey Meat Bird	HPAI 2022	Foam	8/30/2022
00RLDVV	Minnesota	Meeker 06	Commercial Turkey Meat Bird	HPAI 2022	Foam	8/30/2022
00RL07Q	Indiana	Elkhart 04	Backyard Producer	HPAI 2022	CO2/Injectable	8/31/2022
00RKVZX	Minnesota	Hennepin 01	Backyard Producer	HPAI 2022	KEDS	8/31/2022
